# miRVIT: A Novel miRNA Database and Its Application to Uncover *Vitis* Responses to *Flavescence dorée* Infection

**DOI:** 10.3389/fpls.2018.01034

**Published:** 2018-07-17

**Authors:** Walter Chitarra, Chiara Pagliarani, Simona Abbà, Paolo Boccacci, Giancarlo Birello, Marika Rossi, Sabrina Palmano, Cristina Marzachì, Irene Perrone, Giorgio Gambino

**Affiliations:** ^1^Institute for Sustainable Plant Protection, National Research Council of Italy, Turin, Italy; ^2^Viticultural and Enology Research Centre, Council for Agricultural Research and Economics, Conegliano, Italy; ^3^Research Institute on Sustainable Economic Growth, National Research Council of Italy, Turin, Italy

**Keywords:** disease resistance, jasmonate, miRNAs, phytoplasma, target genes, univocal database

## Abstract

Micro(mi)RNAs play crucial roles in plant developmental processes and in defense responses to biotic and abiotic stresses. In the last years, many works on small RNAs in grapevine (*Vitis* spp.) were published, and several conserved and putative novel grapevine-specific miRNAs were identified. In order to reorganize the high quantity of available data, we produced “miRVIT,” the first database of all novel grapevine miRNA candidates characterized so far, and still not deposited in miRBase. To this aim, each miRNA accession was renamed, repositioned in the last version of the grapevine genome, and compared with all the novel and conserved miRNAs detected in grapevine. Conserved and novel miRNAs cataloged in miRVIT were then used for analyzing *Vitis vinifera* plants infected by *Flavescence dorée* (FD), one of the most severe phytoplasma diseases affecting grapevine. The analysis of small RNAs from healthy, recovered (plants showing spontaneous and stable remission of symptoms), and FD-infected “Barbera” grapevines showed that FD altered the expression profiles of several miRNAs, including those involved in cell development and photosynthesis, jasmonate signaling, and disease resistance response. The application of miRVIT in a biological context confirmed the effectiveness of the followed approach, especially for the identification of novel miRNA candidates in grapevine. miRVIT database is available at http://mirvit.ipsp.cnr.it.

**Highlights:** The application of the newly produced database of grapevine novel miRNAs to the analysis of plants infected by *Flavescence dorée* reveals key roles of miRNAs in photosynthesis and jasmonate signaling.

## Introduction

RNA silencing or RNA interference is a pivotal mechanism of gene regulation conserved in a broad range of eukaryotic organisms, including fungi, plants, and animals (Baulcombe, 2002). Small-endogenous RNAs are important effectors of RNA silencing phenomena and are classified into two major categories based on the nature of their RNA precursors: microRNAs (miRNAs) and small-interfering RNAs (Axtell, 2013). miRNAs are single-stranded RNA molecules of approximately 21 nt length generated from endogenous *MIR* genes (Nozawa et al., 2012). The precursors of miRNAs were processed through DICER-LIKE 1 proteins (DCL1) to release a mature miRNA:miRNA^∗^ duplex (Kurihara and Watanabe, 2004). The mature miRNA loaded into a specific ARGONAUTE (AGO)-associated RNA-induced silencing complex (RISC) guides the identification and cleavage of complementary mRNA targets in a sequence-specific manner (Mallory et al., 2008). The miRNA guide strand is generally more abundant than the miRNA star strand (miRNA^∗^) and is responsible for the RISC-mediated target regulation. However, increasing evidence also suggests an association between miRNA^∗^ and AGO proteins exerting relevant biological functions (Liu et al., 2017). miRNAs are involved in several developmental processes, in genome stability maintenance, and in plant adaptation to biotic and abiotic stresses, as reviewed in many studies (Sunkar et al., 2012; Staiger et al., 2013; Borges and Martienssen, 2015).

Among pathogens infecting plants, phytoplasmas, phloem-limited bacteria belonging to the class of *Mollicutes*, cause serious yield and economic losses in many crops (Bendix and Lewis, 2018). In *Vitis vinifera*, *Flavescence dorée* (FD) and *Bois noir* (BN) are associated with grapevine yellows, the most important and damaging phytoplasma-induced diseases in Europe. In particular, the quarantine disease FD is caused by a phytoplasma (FDp) of the elm yellows group (16SrV-C and V-D; Angelini et al., 2001) transmitted in a persistent and propagative manner by the ampelophagous leafhopper *Scaphoideus titanus* Ball (*Hemiptera Cicadellidae*). Typical symptomatology observed in FDp-infected grapevines generally includes downward rolling of leaves, yellowing or reddening of the leaves according to white or red grape varieties, drying of inflorescences and bunches, shortening of internodes, and lack of cane lignification (Caudwell, 1990). In some cases, the spontaneous and stable remission of FD symptoms accompanied by the disappearance of FDp from the plants may occur, giving rise to “recovery” phenomena (Osler et al., 2004; Maggi et al., 2017). Molecular and physiological changes in grapevine infected and recovered by FDp have recently been investigated (Musetti et al., 2007; Gambino et al., 2013; Margaria et al., 2013, 2014; Vitali et al., 2013; Prezelj et al., 2016); nevertheless, the biological mechanisms underlying these processes are not yet fully understood. Although for some plant-phytoplasma systems, the role of miRNAs in disease development appeared clear (Ehya et al., 2013; Gai et al., 2014, 2018; Minato et al., 2014; Shao et al., 2016; Snyman et al., 2017), no data are currently available in the case of FDp–grapevine interaction.

In the last few years, from the first report concerning the identification of novel miRNAs in grapevine (Carra et al., 2009), several works have been published on this topic, and many conserved and putative novel miRNAs were identified. Different species from the *Vitis* genus were analyzed, the most studied being several cultivars of *Vitis vinifera* (Carra et al., 2009; Pantaleo et al., 2010, 2016; Alabi et al., 2012; Belli Kullan et al., 2015; Sun et al., 2015; Paim Pinto et al., 2016; Bester et al., 2017a,b; Pagliarani et al., 2017; Snyman et al., 2017) and the “Summer Black” hybrid of *V. vinifera* ×*Vitis labrusca* (Wang et al., 2011, 2014; Han et al., 2014; Zhao et al., 2017). In addition, some miRNAs derived from *Vitis amurensis* (Wang et al., 2012) and from a powdery mildew-resistant accession of the wild Chinese *Vitis pseudoreticulata* Baihe-35-1 (Han et al., 2016) were characterized as well. Substantially, all grapevine tissues in several developmental stages (Belli Kullan et al., 2015) and from plants subjected to biotic (Alabi et al., 2012; Singh et al., 2012; Pantaleo et al., 2016; Bester et al., 2017a; Snyman et al., 2017) or abiotic stresses were analyzed (Sun et al., 2015; Pantaleo et al., 2016; Pagliarani et al., 2017). Nevertheless, in these studies, there is no uniformity in the classification of the identified novel miRNAs: hundreds of putative novel miRNAs were indicated in different ways, and often the same miRNA was named differently in different works.

In this study, we classified all putative novel miRNAs discovered in grapevine so far, and not deposited in the public database miRBase, into a single database, namely, miRVIT (available at http://mirvit.ipsp.cnr.it), in order to standardize their classification by assigning a new and univocal denomination. The obtained database was then used to analyze nine small RNA libraries produced from leaf midribs collected from FDp-infected (FD), recovered (R), and healthy (H) plants of the highly susceptible *V. vinifera* cv. Barbera. The expression dynamics of conserved, novel miRNAs and their target transcripts associated with FDp infection and/or recovery processes were investigated.

## Materials and Methods

### Construction of a Single Database of Novel miRNAs From Grapevine

The data related to all the novel miRNAs identified so far in grapevine were collected from 18 already published works (Carra et al., 2009; Pantaleo et al., 2010, 2016; Wang et al., 2011, 2012, 2014; Alabi et al., 2012; Singh et al., 2012; Han et al., 2014, 2016; Belli Kullan et al., 2015; Sun et al., 2015; Paim Pinto et al., 2016; Bester et al., 2017a,b; Pagliarani et al., 2017; Snyman et al., 2017; Zhao et al., 2017). Each novel miRNA was aligned using BLAST both against the known miRNAs deposited in miRBase (Kozomara and Griffiths-Jones, 2014) and against all the novel miRNAs retrieved by the different studies above cited. miRNAs already classified by other authors as isomiRs of conserved miRNAs were not considered. Two or more miRNAs were considered to be a single entity, i.e., unique miRNA, when: (i) the miRNA sequences were the same, (ii) they localize in the same position on the genome, and (iii) they had the same precursors. Each novel miRNA was re-named using a “vvi_miC” code followed by the indications “5p” or “3p” according to its location within the precursor sequence. The sequences of miRNA and miRNA^∗^ (when available) were repositioned on the last version of the grape genome 12X V1 (PN40024^1^). Novel miRNAs sharing the same sequences, but with different precursors located in different regions of the grape genome, were indicated with the same name followed by lowercase letters (a, b, c, etc.). Different isoforms (isomiR) of the same novel miRNA shifting a few nucleotides in the same genomic region within the same precursor were indicated as versions V1, V2, etc., of the same novel miRNA.

### Database of miRNA Targets

The target transcripts of all the conserved and novel miRNAs already validated in grapevine through either degradome or 5′ RACE approaches were collected from 11 works (Carra et al., 2009; Pantaleo et al., 2010, 2016; Wang et al., 2012, 2013, 2014; Han et al., 2014; Jiu et al., 2015; Sun et al., 2015; Leng et al., 2017; Zhao et al., 2017). The annotation of the targets previously identified using the old grapevine annotation, referred to as the 8X genome, was updated to the grapevine transcriptome version 12X V1^2^. In addition, target transcripts of novel miRNAs were searched on the degradome library of “Pinot noir” (Pantaleo et al., 2010), and in parallel using the default parameters of the web-based tool StarScan (sRNA Target Scan, Liu et al., 2015). This tool classifies the sRNA-mediated cleavage events in different categories basing on several parameters related to the cleavage of the sequence of the target gene and miRNA, only the three most reliable classes (Z, I, and II) were considered. For each identified target, the annotation 12X V1 was integrated by Gene Ontology (GO) classifications using the Blast2GO tool^3^.

### Plant Material and Experimental Setup

The biological study was performed on plants of the red grape cultivar Barbera (*V. vinifera*) grown in a vineyard located in Cocconato (Piemonte), northwest Italy, and monitored since 2008 for phytoplasma infection (Maggi et al., 2017). FDp and BNp were detected following a phytoplasma enrichment protocol (Marzachì et al., 1999), and the viruses that commonly affect grapevine in Italy were detected by multiplex RT-PCR (Gambino, 2015). Only the plants without both BNp and the most dangerous viruses infecting grapevine were considered for the experimental trial and further divided into three groups: FDp-infected (FD), recovered (R, i.e., plants found positive to FDp infection in the past, but then resulted FDp-negative and symptomless for the last 2 years), and healthy (H). The FDp isolate present in the vineyard belongs to the 16SrV-C subgroup (Maggi et al., 2017). From each group, five plants of the same age and located on neighboring rows in the vineyard were selected, and fully expanded leaves were collected in July 2014. For diseased plants (FD), only canes showing the typical FD symptoms (Caudwell, 1990) were chosen for sampling, and leaf tissues with advanced necrotic areas were excluded. In R and H plants, leaves inserted in the central region of the shoot were randomly collected. Leaf midribs were isolated using a sterile scalpel, immediately frozen in liquid nitrogen and stored at -80°C until RNA extraction and JA quantification. The five plants selected for each category constituted independent biological replicates; three of them were used for small RNA libraries construction, and all the five plants were used for real-time PCR (RT-qPCR) validation assays.

### Small RNA Library Construction and Sequencing

RNA was extracted from leaf midribs using the mirPremier^®^ microRNA Isolation Kit (Sigma-Aldrich, Inc.) following the manufacturer’s instructions. Nine libraries (three biological replicates for each category H, R, and FD) of small RNAs were obtained using the TrueSeq Small RNA Sample Kit (Illumina, San Diego, CA, United States) and were sequenced using the HISeq 2000 Illumina platform by an external service. All data were processed using the UEA small RNA Workbench (Mohorianu et al., 2017) by first removing 3′-adaptor sequences and then filtering out according to the following criteria: (a) low-quality and length (minimum 16 nt; maximum 30 nt); (b) low-complexity, i.e., sequences containing less than three distinct nucleotides were discarded; (c) reads matching to known transfer and ribosomal RNA were discarded; and (d) only reads matching to the *V. vinifera* assembly 12X^1^ were retained. Any sequences without adaptor matches were excluded from further analysis. The miRNA predictions were performed using miRCat (Moxon et al., 2008) on the above mentioned *V. vinifera* assembly. Precursor sequences were then processed using the MFOLD software (v. 2.3^4^; Zuker, 2003) to analyze folding of hairpin secondary structures. Detection of similarities among predicted miRNAs and annotated miRNAs was performed running the BLAST algorithm against both miRBase and our newly produced database.

Bowtie 1.1.2 software^5^ with no mismatch allowed in the alignment was employed to establish accurate miRNA abundance profiles of the nine sequenced samples. Following alignment, the resulting miRNA counts were normalized for differences in sequencing depths to account for the technical differences across samples. Statistically significant differences (*P* < 0.05) in miRNA expression were estimated by applying the Student’s *t-*test between the three possible couples of combinations (R vs. H, FD vs. R; FD vs. H).

Hierarchical clustering (HCL) analysis was then applied using Pearson’s correlation distance and the software MeV v. 4.9^6^, using as input the normalized values of the reads generated for the three categories H, R, and FD.

### Real-Time PCR Analysis

The quantification of miRNA expression levels was carried out by RT-qPCR following the protocol by Shi and Chiang (2005), with the modifications reported in Pantaleo et al. (2016). For target quantification, total RNA was extracted using the Spectrum^TM^ Plant Total RNA extraction kit (Sigma-Aldrich, Inc.) and RT-qPCR assays were performed as reported by Pantaleo et al. (2016). Expression levels of miRNAs and related target genes were quantified after normalization to either 5.8S rRNA and U6 or *VvUBI* and *VvACT* endogenous genes used as internal controls, respectively. Specific primer pairs used in RT-qPCR reactions are listed in Supplementary Table S1. Transcript and miRNA levels were expressed as the mean and standard errors were calculated over five biological replicates.

### Quantification of Jasmonate Content in Leaf Midribs

400 mg of homogenized sample was freeze dried and transferred with 0.5 mL of methanol:water (1:1 *v/v*) acidified with 0.1 % of formic acid in an ultrasonic bath for 1 h. Samples were centrifuged for 2 min at 4°C and 15,000 rpm, and the supernatant was analyzed by high-performance liquid chromatography (HPLC). Original standard of (±)-jasmonic acid (purity ≥ 95%, Sigma-Aldrich) was used for the identification by comparing retention time and UV spectrum. The quantification was made by external calibration method.

The HPLC apparatus was an Agilent 1220 Infinity LC system (Agilent^®^, Waldbronn, Germany) model G4290B equipped with gradient pump, auto-sampler, and column oven set at 30°C. A 170 Diode Array Detector (Gilson, Middleton, WI, United States) set at 280 nm was used as detector. A XTerra RP18 analytical column (150 mm × 6 mm i.d., 5 μm, Waters) was used. The mobile phases consisted in water acidified with formic acid 0.05% (A) and acetonitrile (B), at a flow rate of 0.500 mL min^-1^ in gradient mode, 0–20 min: from 10 to 35% of B, 20–25 min: from 35 to 100% B; 20 μL was injected for each sample.

### Statistical Analyses

Significant differences among treatments were statistically analyzed by applying a one-way ANOVA test followed by the Tukey’s *HSD post hoc* test (*P ≤ 0.05*). Significant differences of pairwise comparisons were assessed by Student’s *t*-test. The SPSS statistical software package (SPSS Inc., Cary, NC, United States, v.23) was used to run statistical analyses.

### Accession Numbers

Raw sequences from the nine miRNA libraries were deposited at the NCBI Sequence Read Archive under the accession number SRP129862.

## Results and Discussion

### miRVIT, a Novel Grapevine miRNA Database

Using the data reported in 18 works published until now on miRNAs in the *Vitis* genus, all putative novel miRNAs (hereafter referred to as novel miRNAs) identified and still not deposited in miRBase were collected into a single database, called “miRVIT” (available at http://mirvit.ipsp.cnr.it, Supplementary Table S2). Overall, 901 sequences referred to as novel miRNAs, re-named using a “vvi_miC” code, were found; among these, 22 are miRNAs or miRNAs^∗^ already deposited in miRBase and were excluded. In some cases, the same novel miRNA was reported using different classifications in more than 10 different works (Supplementary Table S2). For instance, 14 research groups identified vvi_miC1039, 13 identified vvi_miC132 (Supplementary Figure S1), and 11 identified vvi_miC137. The sequences of precursors were obtained by bioinformatics predictions, and different software or different versions of the same software were employed depending on the year of publication of the previous works, using in some cases different version of the grapevine genome. Consequently, some differences in the precursor sequences outside the region between miRNA and miRNA^∗^ were identified for the same novel miRNA (Supplementary Figure S1).

**FIGURE 1:**
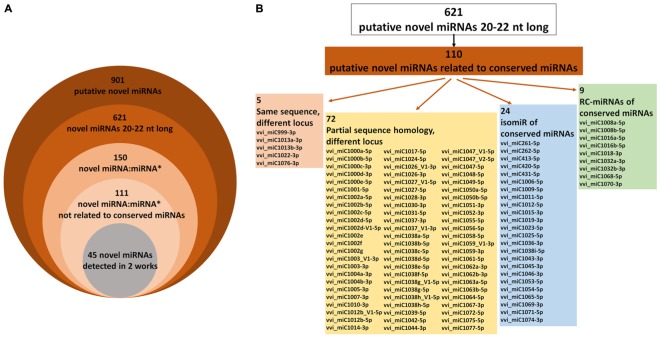
**(A)** The numbers of novel miRNAs detected so far in grapevine and the subgroups in which the miRVIT database was divided according to the length of small RNAs, the presence of miRNA:miRNA^∗^ duplex, and similarity with conserved miRNAs. **(B)** Schematic subdivision of novel miRNAs showing some sequence relations with conserved miRNAs.

Such a large number of putative grapevine-specific novel miRNAs do not appear realistic based on current knowledge, as it is conceivable that many false positives are present in this list, since in *Arabidopsis* (the most intensively studied plant species), only a few hundred miRNAs are currently known (Taylor et al., 2017; Axtell and Meyers, 2018). Starting from the criteria published for plant miRNA annotation (Meyers et al., 2008; Axtell and Meyers, 2018), we considered as novel miRNAs only the 20–22 nt long small RNAs, thus excluding small RNA sequences of all other lengths. Indeed, heterochromatic siRNAs of 23–24 nt are very common in small RNA-seq libraries, while miRNAs of 23–24 nt in length are rare and require extremely strong evidence to be classified as miRNAs (Axtell and Meyers, 2018), generally not available for grapevine novel miRNAs. The remaining 621 miRNAs 20–22 nt long were classified as 469 unique novel miRNAs (**Figure 1A** and Supplementary Table S3). The largest families of these novel miRNAs are vvi_miC44-5p, with 10 members, identified by Pantaleo et al. (2010) and partially by Han et al. (2016), and vvi_miC1038-5p, counting nine members and two isomiRs identified in several works (Supplementary Table S3). It must be taken into account that one-sixth of these novel miRNAs (110 on 621) showed either sequence similarity or some relationship with conserved miRNAs already deposited in miRBase (**Figure 1B** and Supplementary Table S3). This is the case of vvi_miC999-3p, vvi_miC1013, vvi_miC1022, and vvi_miC1076, which share the same sequences with vvi-miR3635-3p, vvi-miR171, vvi-miR390, and vvi-miR3634-3p, respectively, although they are located in different loci. Moreover, 72 novel miRNAs showed sequence similarity with several conserved miRNAs (particularly vvi-miR535, vvi-miR477, and vvi-miR3631), although they originated from different precursors at different genomic locations (Supplementary Table S3). Twenty-four of these are isomiRs of conserved miRNAs, which may have been not correctly classified in the original works, and nine are reverse-complementary miRNAs (RC-miRNAs, generated from the antisense strands of the miRNA precursors) of conserved miRNAs or miRNAs^∗^ (**Figure 1B** and Supplementary Table S3). In addition, other RC-miRNAs were detected in three pairs of novel miRNAs not related to conserved miRNAs: vvi_miC265-5p/vvi_miC266-5p, vvi_miC343-5p/vvi_miC344, and vvi_miC427a/vvi_miC427b. RC-miRNAs, originally detected in animals (Tyler et al., 2008), were rarely described in plants (Shao et al., 2012; Belli Kullan et al., 2015), and their biological function is not yet completely defined, even though a potential role in transcriptional regulation through a DNA methylation-mediated pathway was hypothesized (Shao et al., 2012).

Of the 621 novel miRNAs analyzed, miRNA^∗^ sequences were identified for only 150 accessions, and 45 of these were found in at least two different works, e.g., in at least two different small RNA libraries originating from different genotypes or tissues (Axtell and Meyers, 2018). Hypothetically, only 5% (45 of 901) of the novel miRNAs previously identified in grapevine satisfied the stringent requirements and could be really considered as novel miRNA candidates (**Figure 1A** and Supplementary Table S4). However, in the following sections, all the 621 20–22 nt long novel miRNAs were analyzed and their functional role was studied in a biological experiment to validate the incidence of these miRNA candidates in *V. vinifera* cv. Barbera.

### Target Genes of Grapevine miRNAs

Besides novel miRNAs, the miRVIT database groups all the target genes of conserved and novel miRNAs validated so far in grapevine using either degradome sequencing or 5′ RACE. Overall, 215 targets of conserved miRNAs and 113 targets of novel miRNAs were reported in Supplementary Table S5. In addition, the database was integrated by searching target transcripts of novel miRNAs (Supplementary Table S3) in the degradome library of “Pinot noir” (Pantaleo et al., 2010) using the web-based tool StarScan (sRNA Target Scan, Liu et al., 2015). A similar analysis was previously validated by confirming the degradome predictions through 5′ RACE (Pantaleo et al., 2016), thus proving to be a more reliable approach than bioinformatics prediction alone. Using StarScan, 213 potential targets (seven of which were previously validated, see Supplementary Table S5) of 120 novel miRNAs were identified (Supplementary Table S6). Noteworthy, the novel miRNAs related to conserved miRNAs (**Figure 1B**) generally targeted the same transcripts of the conserved miRNA. For example, the targets of vvi_miC1026 and vvi_miC1027 that have sequences similar to vvi-miR396 are a number of transcripts encoding growth-regulating factors, whereas vvi_miC1031-5p (similar to vvi-miR403) targeted *VvAGO2* and vvi_miC1000 (similar to vvi-miR156) targeted mRNAs encoding Squamosa promoter-binding proteins (Supplementary Table S6).

### Small RNA Sequencing and Identification of miRNAs in “Barbera” Leaf Midribs

After removing adapters, redundant and low-quality sequences, reads from nine small RNA libraries of “Barbera” leaf midribs ranging from 16 to 30 nucleotides (see the metrics of the libraries in Supplementary Table S7 and Supplementary Figure S2) were aligned onto sequences of miRNAs deposited in miRBase and against miRVIT (Supplementary Table S3). As expected, we identified members of almost all known miRNAs: 137 of 168 (73.6%) deposited in miRBase. The normalized reads of each conserved miRNA were reported in Supplementary Table S8, showing that several members of vvi-miR166 family and vvi-miR3634-3p were the most expressed in “Barbera” leaf midribs. Of the 621 novel miRNAs previously identified in grapevine tissues, 308 were expressed at least in one “Barbera” small RNA library, and in 67 cases both miRNA and miRNA^∗^ were identified (Supplementary Table S9). Interestingly, the probability of detecting previously published novel miRNAs in “Barbera” was associated to specific factors, such as tissue-type, stress conditions, and genotype from which the novel miRNAs were originally isolated. In particular, 73.1% of the novel miRNAs originally isolated from either leaf or phloem tissues of grapevine (Alabi et al., 2012; Singh et al., 2012; Sun et al., 2015; Han et al., 2016; Pantaleo et al., 2016; Bester et al., 2017a,b; Snyman et al., 2017) were also detected in “Barbera” leaf midribs; on the contrary, only 53.8% of the novel miRNAs previously characterized in different organs and tissues (Carra et al., 2009; Pantaleo et al., 2010; Wang et al., 2011, 2012, 2014; Han et al., 2014; Belli Kullan et al., 2015; Paim Pinto et al., 2016; Pagliarani et al., 2017; Zhao et al., 2017) were found in the sequenced samples. Furthermore, 76.3% of the novel miRNAs identified in grapevine plants subjected to virus or phytoplasma infection (Alabi et al., 2012; Pantaleo et al., 2016; Bester et al., 2017a; Snyman et al., 2017) were also retrieved in FDp-infected samples of “Barbera” (Supplementary Table S9). Gene expression is notoriously influenced by tissue and environmental conditions; consequently, the probability to identify a miRNA in a small RNA library can be affected by these conditions as well. The genotype effect appeared more complicated to be clarified, as the database was biased to a higher number of works published on *V. vinifera* (Carra et al., 2009; Pantaleo et al., 2010, 2016; Alabi et al., 2012; Belli Kullan et al., 2015; Sun et al., 2015; Paim Pinto et al., 2016; Bester et al., 2017a,b; Pagliarani et al., 2017; Snyman et al., 2017) than on *V. amurensis* (Wang et al., 2012) or on *V. pseudoreticulata* (Han et al., 2016). While it was evident the low probability to identify in “Barbera” novel miRNAs originally published in *V. amurensis* (Wang et al., 2012), we did not observe a marked difference among novel miRNAs originally uncovered in *V. vinifera*, its hybrids, or *V. pseudoreticulata* (Supplementary Figure S3). Currently, the identification of novel miRNA loci in different *Vitis* spp. was biased to the use of PN40024 (Jaillon et al., 2007) or “Pinot noir” (Velasco et al., 2007) reference genomes, which show potential genomic differences relying on the cultivars or on other *Vitis* species in which the miRNAs were detected (Morgante et al., 2007; Gambino et al., 2017). Consequently, the hairpin prediction and the mapping of the reads produced for instance from *V. pseudoreticulata* on the “Pinot noir” genome (Han et al., 2016) allowed the prediction of only the miRNA loci common to *V. vinifera* cv. Pinot noir. The species-specific reads not mapping on the reference genome were generally discarded. This could be an explanation of the unexpected elevated percentage of miRNAs originally identified in *V. pseudoreticulata* and detected in “Barbera” (Supplementary Figure S3). In the near future, whether new *Vitis* genomes became available, it would be interesting to re-analyze small RNA libraries already published, in order to identify potential new species- or cultivar-specific miRNAs.

### Putative Novel miRNAs Identified for the First Time in “Barbera”

In addition to the novel miRNAs previously identified and annotated in miRVIT (Supplementary Table S2), other novel miRNA candidates were characterized for the first time in “Barbera” using the miRCat tool and the latest release of the *V. vinifera* genome. Excluding the miRNAs already present in miRBase and miRVIT, and considering only the 20–22 nt long miRNAs and those expressed in at least three different libraries with at least 10 non-normalized reads, 13 putative novel miRNAs were selected (hereafter called “Barbera”-novel miRNAs; **Table 1** and Supplementary Figure S4). In addition, four variants of novel miRNAs already included in miRVIT were identified. However, no miRNA^∗^ sequences were found for “Barbera”-novel miRNAs, and no significant candidate target genes were retrieved starting from the degradome library of “Pinot noir” (Pantaleo et al., 2010) and using the StarScan tool.

**Table 1:** Putative novel miRNAs identified for the first time in “Barbera” leaf midribs (“Barbera” – novel miRNAs).

miRNA ID	Genomic position	Orientation	Sequence (5′–3′)	sRNA length (nt)	Hairpin length	Hairpin minimum free energy (ΔG kcal mol^-1^)	miRNA^∗^ and Notes
vvi_miC134_V1-3p	chr5: 5125249 5125229	-	CTGAGGCTTCATTTTTGAACT	21	114	-61.50	Variant of vvi_miC134-3p in the same locus.
vvi_miC134_V1b-3p	chr15: 14464031 14464051	+	CTGAGGCTTCATTTTTGAACT	21	113	-37.70	Same sequences of vvi_miC134_V1-3p, different genomic location.
vvi_miC443_V1-3p	chr18: 3551253 3551273	-	AAATTGGCTCTGTAAATTTCT	21	142	-79.68	vvi_miC443_V1-5p: AATATTACAGAGCCATTTTGG (chr18: 3551368 3551348) Variants of vvi_miC443 in the same locus.
vvi_miC593_V1-5p	chrUn: 26025545 26025524	-	TGGTGAACCAAATAACTCTGGT	22	159	-63.60	vvi_miC593_V1-3p: ACCAGAGTTCTTTAGTTGACG (chrUn: 26025482 26025462) Variants of vvi_miC593 in the same locus.
vvi_miC597-3p	chr2: 636221 636241	-	CCAGGATTCGAACTCAAGACC	21	118	-59.75	In the same locus and partially complementary to vvi_miC49-5p
vvi_miC605-3p	chr10: 10801472 10801491	+	AGAGGCTCGGTGAAATAGAC	20	98	-27.10	
vvi_miC606-3p	chr10: 2522406 2522427	-	GCTAGCAAATGGAGTCCTGAAC	22	85	-24.40	
vvi_miC614-5p	chr14: 21339372 21339392	-	TTTAGGAAGTGTTTCTGGGCT	21	121	-65.90	
vvi_miC617-3p	chr14: 27797794 27797814	-	TCCCAACAAAAACGTCGGCCT	21	158	-74.30	
vvi_miC620-3p	chr16: 286248 286268	-	CCGACGTTACGCTTGAGCAGT	21	129	-63.40	
vvi_miC630-3p	chr19: 3919987 3920007	+	CTTGGTTCCTGGAAAATTTGA	21	156	-50.76	
vvi_miC631-3p	chr2: 15941291 15941311	-	ATGTTATGGATCTTGGTTTGT	21	94	-24.20	
vvi_miC638-3p	chr4: 1959986 1960005	+	GGAAGAGCTAGAATTCTAAC	20	63	-18.80	
vvi_miC644-3p	chr6: 8570952 8570972	+	TTGAGCGATCAAAACGGGCCT	21	118	-32.18	
vvi_miC645-5p	chr7: 1677361 1677381	+	AGAGGAACCGAAACTAGGACT	21	120	-70.60	
vvi_miC648-5p	chr7: 3062319 3062339	-	TTAAGCGAGACCATTGTGACT	21	81	-22.50	
vvi_miC653-5p	chr9: 328798 328818	+	TCAACTATGAGCCCCTTGAAT	21	117	-43.10	

### Expression Profiles of miRNAs in “Barbera” Leaf Midribs

**FIGURE 2:**
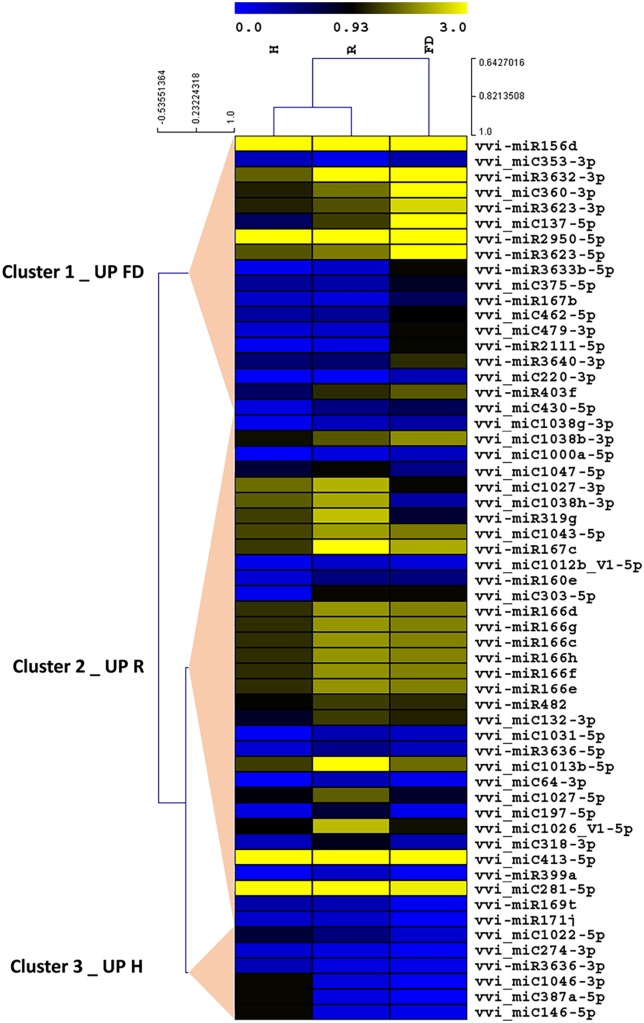
Hierarchical clustering analysis of conserved and novel miRNAs differentially modulated among FDp-infected (FD), recovered (R), and healthy (H) leaf midribs of “Barbera” (*p* ≤ 0.05). The clustering was generated with the TMeV software (v. 4.9) using the average normalized number of reads of three biological replicates. The results were represented by heat map blue–yellow corresponding, respectively, to low and high miRNAs accumulation levels.

Despite the high number of miRNAs expressed in our libraries, only 25 conserved (**Table 2**) and 32 novel miRNAs (**Table 3**) showed significant differences in at least one comparison among the three considered theses (FD vs. H, R vs. H, and FD vs. R). A possible explanation for this could rely on the highly variable response of the different biological replicates constituting single “Barbera” plants. Nevertheless, by following this approach, the significant expression differences investigated in this work should be more reliable than those obtained with other sampling methodologies. An HCL analysis involving these 57 miRNAs was conducted to investigate the relationships of similarity among the three experimental categories. H and R formed a separate clade from FD, and the miRNAs were clustered in three groups: Cluster 1 included miRNAs with higher accumulation in FD, Cluster 2 counted miRNAs showing a slight peak of expression in R, and Cluster 3 contained only six miRNAs with higher expression in H (**Figure 2**).

**Table 2:** Normalized number of reads (mean of three biological replicates) of conserved miRNAs in leaf midribs of FDp infected (FD), recovered (R), and healthy (H) “Barbera.”

Conserved miRNA	Normalized number of reads			Significant differences		
	H	R	FD	FD/H	R/H	FD/R
vvi-miR156d	4.76	4.12	5.51		^∗^	
vvi-miR160e	0.15	0.47	0.47		^∗^	
vvi-miR166c	13119.96	21411.64	19961.37		^∗^	
vvi-miR166d	13161.60	21517.84	19907.53		^∗^	
vvi-miR166e	12980.16	21368.43	19904.17		^∗^	
vvi-miR166f	13019.97	21318.63	19943.36		^∗^	
vvi-miR166g	13058.51	21486.03	19877.01		^∗^	
vvi-miR166h	13065.48	21401.90	19950.53		^∗^	
vvi-miR167b	0.35	0.38	0.93	^∗^		^∗^
vvi-miR167c	1.40	3.09	2.32		^∗^	
vvi-miR169t	0.00	0.11	0.03		^∗^	
vvi-miR171j	0.00	0.11	0.07		^∗^	
vvi-miR319g	1.44	2.50	0.75			^∗^
vvi-miR399a	0.03	0.20	0.03		^∗^	^∗^
vvi-miR403f	20.99	32.73	41.89	^∗^		
vvi-miR482	96.98	143.93	128.36		^∗^	
vvi-miR2111-5p	0.56	1.28	1.66	^∗^		
vvi-miR2950-5p	3.17	6.80	21.33	^∗^		^∗^
vvi-miR3623-3p	120.73	159.46	267.45	^∗^		^∗^
vvi-miR3623-5p	81.04	96.61	181.92	^∗^		^∗^
vvi-miR3632-3p	1.72	3.20	5.94	^∗^		
vvi-miR3633b-5p	0.38	0.32	0.80	^∗^		^∗^
vvi-miR3636-3p	0.18	0.19	0.03	^∗^		
vvi-miR3636-5p	0.17	0.42	0.24		^∗^	
vvi-miR3640-3p	0.12	0.46	0.62	^∗^		

*The asterisk denotes significant differences in the three comparisons FD/H, R/H, and FD/R using the Student’s t-test (P ≤ 0.05).*

**Table 3:** Normalized number of reads (mean of three biological replicates) of novel miRNAs in leaf midribs of FDp infected (FD), recovered (R), and healthy (H) “Barbera.”

Putative novel miRNA	Normalized number of reads			Significant differences		
	H	R	FD	FD/H	R/H	FD/R
vvi_miC64-3p	0.03	0.27	0.08		^∗^	
vvi_miC132-3p	15.62	28.48	24.17	^∗^	^∗^	
vvi_miC137-5p	0.59	1.43	3.83	^∗^	^∗^	^∗^
vvi_miC146-5p	0.27	0.11	0.08	^∗^		
vvi_miC197-5p	0.10	0.72	0.07		^∗^	^∗^
vvi_miC220-3p	0.05	0.25	0.34		^∗^	
vvi_miC274-3p	0.32	0.28	0.05			^∗^
vvi_miC281-5p	0.00	0.14	0.03		^∗^	^∗^
vvi_miC303-5p	0.07	0.00	0.00	^∗^	^∗^	
vvi_miC318-3p	0.25	0.83	0.29		^∗^	
vvi_miC353-3p	0.25	0.08	0.31		^∗^	
vvi_miC360-3p	1.19	1.86	3.75	^∗^		
vvi_miC375-5p	0.20	0.14	0.60	^∗^		^∗^
vvi_miC387a-5p	0.17	0.11	0.03	^∗^		
vvi_miC413-5p	3.02	5.27	3.11		^∗^	^∗^
vvi_miC430-5p	0.02	0.12	0.23	^∗^		
vvi_miC462-5p	0.52	0.53	1.29	^∗^		
vvi_miC479-3p	0.03	0.03	0.26	^∗^		^∗^
vvi_miC1000a-5p	0.05	0.11	0.00			^∗^
vvi_miC1012b_V1-5p	0.07	0.20	0.13			^∗^
vvi_miC1013b-5p	42.82	91.15	54.42		^∗^	
vvi_miC1022-5p	4.95	4.75	2.87	^∗^		
vvi_miC1026_V1-5p	0.93	2.45	1.07		^∗^	
vvi_miC1027-3p	1.81	2.39	0.98	^∗^		^∗^
vvi_miC1027-5p	8.76	16.95	7.65		^∗^	^∗^
vvi_miC1031-5p	0.03	0.28	0.21		^∗^	
vvi_miC1038b-3p	0.15	0.20	0.00			^∗^
vvi_miC1038g-3p	0.07	0.20	0.00			^∗^
vvi_miC1038h-3p	1.68	2.30	0.34			^∗^
vvi_miC1043-5p	150.00	222.32	194.43		^∗^	
vvi_miC1046-3p	0.72	0.48	0.18	^∗^	^∗^	
vvi_miC1047-5p	7.19	9.83	4.46			^∗^

*The asterisk denotes significant differences in the three comparisons FD/H, R/H, and FD/R using the Student’s t-test (P ≤ 0.05).*

**FIGURE 3:**
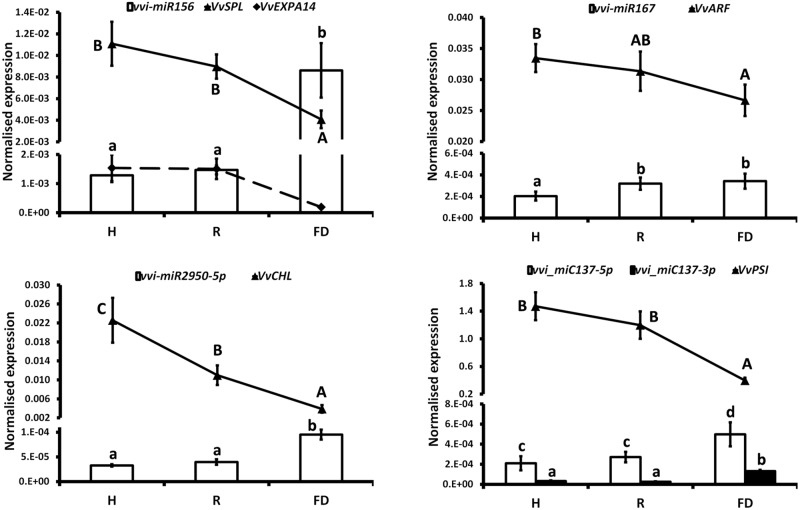
Expression levels of vvi-miR156, vvi-miR167, vvi-miR2950-5p, vvi_miC137-5p, and their respective target transcripts, *VvSPL* (VIT_01s0010g03910), *VvEXPA14* (VIT_13s0067g02930), *VvARF* (VIT_12s0028g01170), *VvCHL* (VIT_07s0151g00250), and *VvPSI* (VIT_05s0020g03180), in FDp-infected (FD), recovered (R), and healthy (H) leaf midribs of “Barbera.” qRT-PCR signals were normalized to U6 and 5.8 rRNA for miRNA quantification, and to actin and ubiquitin transcripts for target quantification. Lowercase and uppercase letters denote significant differences (*p* ≤ 0.05) among miRNAs and target expression levels, respectively, tested using Tukey’s HSD test. Data are presented as mean ± standard error of five biological replicates (*n* = 5).

**FIGURE 4:**
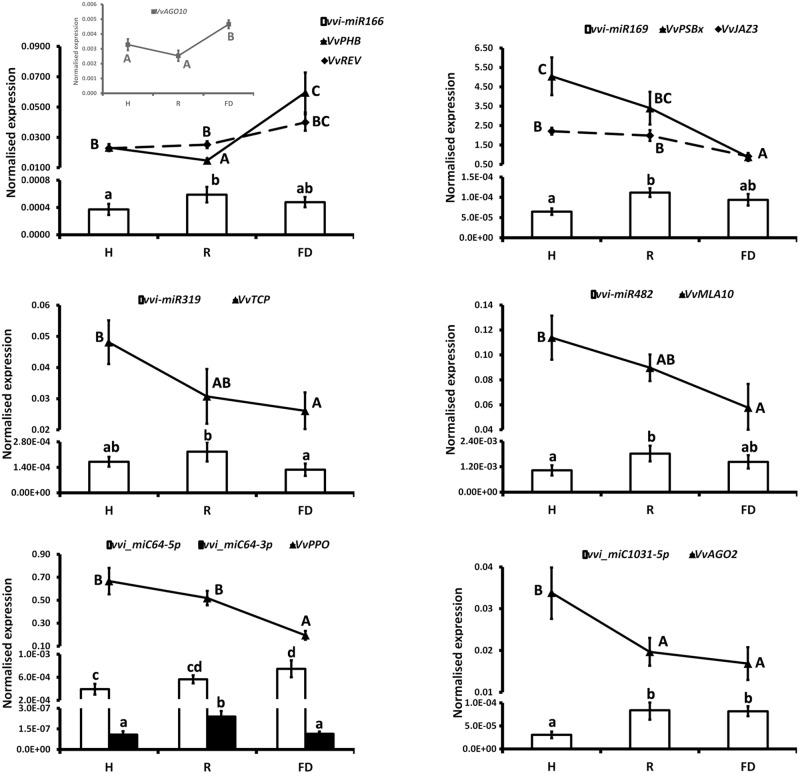
Expression levels of vvi-miR166, vvi-miR169, vvi-miR319, vvi-miR482, vvi_miC1031-5p, vvi_miC64, and their respective target transcripts, *VvPHB* (VIT_10s0003g04670), *VvREV* (VIT_13s0019g04320), *VvPSBx* (VIT_04s0008g01730), *VvJAZ3* (VIT_01s0011g05560), *VvTCP* (VIT_12s0028g02520), *VvMLA10* (VIT_04s0023g02380), *VvAGO2* (VIT_10s0042g01180), *VvPPO* (VIT_10s0116g00560), and *VvAGO10* (VIT_05s0020g04190, inset), in FDp-infected (FD), recovered (R), and healthy (H) leaf midribs of “Barbera.” qRT-PCR signals were normalized to U6 and 5.8 rRNA for miRNA quantification, and to actin and ubiquitin transcripts for target quantification. Lowercase and uppercase letters denote significant differences (*p* ≤ 0.05) among miRNAs and target expression levels, respectively, tested using Tukey’s HSD test. Data are presented as mean ± standard error of five biological replicates (*n* = 5).

The expression changes of the most interesting miRNAs (**Tables 2**, **3**) and their already validated target genes (Supplementary Table S5) were further investigated by RT-qPCR. In Cluster 1, the higher accumulation of miRNAs induced by FDp infection was confirmed for vvi-miR156, vvi-miR167, vvi-miR2950-5p, and vvi_miC137 (**Figure 3**) and for vvi-miR3632-3p, vvi-miR3623-5p, vvi-miR403, vvi_miC360-3p, and vvi_miC430-3p (Supplementary Figure S5). In addition, RT-qPCR assays conducted on the miRNAs belonging to Cluster 2, vvi-miR166, vvi-miR169, vvi-miR319, vvi-miR482, vvi_miC1031-5p, vvi_miC64 (**Figure 4**), vvi_miC1038-3p, vvi_miC132-3p, vvi_miC197-5p, vvi_miC281-5p, and vvi_miC413-5p (Supplementary Figure S6), confirmed the expression trends observed in sequencing results. The “Barbera”-novel miRNAs were expressed in all groups of the considered experimental plan, and for six of them, significant differences were observed among the treatments (Supplementary Table S10). The RT-qPCR analyses conducted on a selection of these miRNAs, vvi_miC606-3p, vvi_miC617-3p, vvi_miC644-3p, and vvi_miC648-5p, were consistent with sequencing data, and vvi_miC644-3p, which was the most expressed among the “Barbera”-novel miRNAs, was overexpressed in FD samples (Supplementary Figure S7). Further analyses are needed to prove their biological functions definitely. Indeed, the biological effect of miRNAs is generally mediated by their cleavage activity on specific target genes.

### Dissecting the Role of miRNAs in the Regulation of the FDp–Grapevine Interaction

miRNAs and target transcripts significantly modulated in the presence of FDp influenced the metabolic processes linked to photosynthesis, cellular development, jasmonate (JA) signaling, and defense responses. The reduction of photosynthesis efficiency caused by FDp infection, which was previously suggested by physiological experiments (Vitali et al., 2013), was likely induced by the down-regulation of specific genes, such as the photosystem I reaction center subunit II (*VvPSI*, VIT_05s0020g03180) and the Photosystem II subunit X (*VvPSBx*, VIT_04s0008g01730), encoding important proteins of the Photosystems I and II reaction centers, which were targeted by vvi_miC137-3p and vvi-miR169, respectively (**Figures 3**, **4**). These results were consistent with the observed downregulation of a chlorophyllase-encoding gene (*VvCHL*, VIT_07s0151g00250), targeted by the grape specific miRNA vvi-miR2950 and influencing chlorophyll catabolism in infected plants (**Figure 3**). Accordingly, both vvi-miR2950 and the novel vvi_miC137-3p were highly induced upon FDp infection, showing a modulation pattern that was negatively correlated to the photosynthetic efficiency measured in diseased grapevines (Pantaleo et al., 2016).

Among highly conserved miRNA categories involved in plant development, miR156 is a well-known regulator of Squamosa promoter-binding protein-like genes (*SPL*, Wu and Poethig, 2006; Ferreira e Silva et al., 2014). The SPL transcription factor family exerts a key role in controlling the transition of floral phases, plant architecture, and in the determination of leaf cell number and size (Chen et al., 2010). In grapevine, the *VvSPL* gene (VIT_01s0010g03910) is a target of vvi-miR156 (Pantaleo et al., 2010), together with an expansin-encoding gene (*VvEXPA14*, VIT_13s0067g02930, Wang et al., 2014), involved in cell wall modification under stress conditions (Dal Santo et al., 2013). Our results indicated that FD presence strongly inhibited these transcripts, differently from what observed for the expression trend of vvi-miR156 upon infection (**Figure 3**). It is thus conceivable that the FD-mediated activation of vvi-miR156 with the consequent repression of *SPL* genes could be at the base of the developmental alterations (i.e., shortening of internodes, smaller inflorescences, etc.) that are typical symptoms associated to FDp infection in grapevine (Caudwell, 1990). These data are also supported by previous evidences attesting that, besides being a master regulator of plant development, vvi-miR156 is able to dynamically respond to environmental stresses in different ways (Alabi et al., 2012; Sun et al., 2015; Pantaleo et al., 2016; Pagliarani et al., 2017). The strong upregulation here observed in FDp infected leaves is in contrast with data reported in grapevines infected by aster yellows (AY; Snyman et al., 2017). Nevertheless, in the associations of *Ziziphus jujuba*/Jujube witches’-broom (Shao et al., 2016), Mexican lime (*Citrus aurantifolia* L.)/Witches’ broom disease (Ehya et al., 2013) and mulberry (*Morus multicaulis* Perr.)/mulberry yellow dwarf disease (Gai et al., 2014), miR156 was always overexpressed in infected plants, as in our study, thus highlighting its potential function in directing symptom development.

Another family of miRNAs typically tied to plant development is miR166, which regulates the broad class of III homeodomain leucine zipper (HD-ZIPIII) transcription factors. In particular, these genes were associated with *Arabidopsis* embryo and meristem development, organ polarity, and vascular development (Baucher et al., 2007 and the references therein). The levels of vvi-miR166 in FDp infected plants seemed not to justify the high transcriptional rates of *Phabulosa* (*VvPHB*, VIT_10s0003g04670) and *Revoluta* (*VvREV*, VIT_13s0019g04320) transcripts (**Figure 4**). A possible explanation could be linked to a direct activation of the promoters of these genes in response to FDp that may escape the miRNA-mediated regulation, as also suggested for other miRNA–target interactions in grapevine (Pantaleo et al., 2016; Pagliarani et al., 2017). In addition, it must be considered that the complex regulatory system “AGO10-mir166-HD-ZIPIII” uncovered in *Arabidopsis*, in which AGO10 specifically sequesters miR166 and antagonizes the silencing activity mediated by AGO1-miR166 against HD-ZIP III (Zhou et al., 2015), may also occur in grapevine. This hypothesis is consistent with the observed overexpression of *VvAGO10* (VIT_05s0020g04190) in FDp infected plants (inset in **Figure 4**), which could reduce the post-transcriptional activity of vvi-miR166 with a consequent increase in *VvPHB* and *VvREV* expression levels. In addition to development regulation, REV was also involved in leaf senescence in *Arabidopsis* through the direct transcriptional induction of a WRKY transcription factor (Xie et al., 2014). The homologous WRKY gene in grapevine, *VvWRKY1*, was overexpressed in FD-infected samples (Gambino et al., 2013), in agreement with a potential role as trigger of defense responses by causing a decrease in the plant susceptibility to the pathogen presence, as indicated for some fungi (Marchive et al., 2013). The activity of *VvWRKY1* in grapevine against fungi, and hypothetically against FDp, involved the activation of distinct sets of defense-related genes. In particular, in grapevine infected by FDp, salicylic acid (SA)-mediated signaling activation and JA repression were previously suggested (Gambino et al., 2013; Prezelj et al., 2016). This type of regulation was confirmed by the present work showing that could be influenced by the activity of some miRNAs. The alteration of JA biosynthetic pathway could be promoted by tuning the TEOSINTE BRANCHED/CYCLOIDEA/PCF (*VvTCP*, VIT_12s0028g02520)/miR319 interplay (**Figure 4** and Supplementary Figure S7) through the downregulation of JA ZIM domain-containing protein (*VvJAZ3*, VIT_01s0011g05560), target of both vvi-miR169 and the novel vvi_miC197-5p (**Figure 4**), and an auxin response factor-encoding transcript (*VvARF*, VIT_12s0028g01170), target of vvi-miR167 (**Figure 3**). The downregulation of *VvTCP* occurred almost exclusively in FDp-infected plants and in parallel with the significant reduction of both *VvLOXA* and 12-oxophytodienoate reductase (*VvOPR3*) expression (Supplementary Figure S8), two of the most important genes involved in JA biosynthesis (Wasternack and Hause, 2013). Interestingly, the interplay *VvTCP*/miR319 controls the biosynthesis of JA through the regulation of lipoxygenase (*LOX*) genes (Schommer et al., 2008). Consistently, the quantification of JA content in the same tissues displayed a progressive reduction or even absence of this metabolite in R and FD samples, respectively (Supplementary Figure S8).

The ARF/miR167 system is involved in the regulation of auxin responsive genes influencing both auxin signaling and JA pathway (Gutierrez et al., 2012; Boer et al., 2014, and the references therein). Consequently, in FDp-infected grapevine, the *VvARF*/vvi-miR167 regulation, associated to the observed decrease in JA content, could be the base of the flower alterations typical of infected plants, in agreement with other reports showing that the overexpression of miR167 in tomato induces floral developmental defects in a JA-dependent manner (Liu et al., 2014). Moreover, a clear correlation among auxin, JA, and phytoplasma was previously demonstrated in transgenic *Arabidopsis* plants containing the phytoplasma virulence effector tengu-su inducer (TENGU) of “*Candidatus* Phytoplasma asteris,” onion yellows isolate (Hoshi et al., 2009). These *Arabidopsis* plants showed high levels of miR167 and a decreased expression of both *ARF* and *LOX* genes associated to symptom developments and reduction in JA and auxin levels (Minato et al., 2014). Similar results were obtained using transgenic *Arabidopsis* expressing the AY-WB protein 11 (SAP11), an effector produced by the Aster Yellows Witches’ Broom (AY-WB) phytoplasma (Sugio et al., 2011). However, repression of JA pathway is not a generic response to phytoplasma infection, as in BNp-infected grapevines very different molecular and metabolic responses were reported. Indeed, JA biosynthesis was strongly activated in both BNp-recovered and diseased grapevines over the whole vegetative season (Paolacci et al., 2017). In parallel, the activation of the SA signaling pathway was also observed, as previously reported (Hren et al., 2009: Dermastia et al., 2015). The authors hypothesized an SA-mediated repression of the genes downstream the JA biosynthesis with relative inhibition of the JA signaling in infected plants. Additionally, the activation of JA-mediated pathway in recovered grapevines could support the role of this defense pathway in the maintenance of the recovery condition in former BNp-infected grapevines (Paolacci et al., 2017). Although this hypothesis may be interesting, it could be valid only for the BNp, as FDp induces very different responses in plant (i.e., induction of SA and JA repression); thus, further insights on the specific FDp-grapevine pathosystem will be necessary to investigate this point in depth.

The regulation of miRNAs and targets linked to disease resistance induced in some cases a reduction of these responses in FDp infected plants. For example, miR482 is involved in a feedback control of NBS-LRR genes in several species, lowering the energy cost of disease resistance process by downregulating the production of these genes in the absence of pathogens (González et al., 2015). In FDp-infected grapevines, we observed an opposite regulation of this system with reduction of *VvMLA10* (VIT_04s0023g02380) in the presence of the pathogen (**Figure 4**). A similar downregulation was noticed for *VvAGO2* (VIT_10s0042g01180) in FD samples. In grapevine, this gene is targeted by the novel vvi_miC1031-5p (with a sequence similar to vvi-miR403) and likely by vvi-miR403, as observed in other plants (Harvey et al., 2011), although this has not been confirmed in grapevine yet. AGO2 has an antiviral role against several viruses in different species (Harvey et al., 2011), while its relationship with phytoplasma infection is not currently clear. The downregulation of AGO2 here reported in FD condition (**Figure 4**) could suggest that phytoplasma-infected plants may be more susceptible to virus infection compared to healthy ones. This hypothesis was not confirmed in BNp infected plants, where the presence of phytoplasma did not influence the virus infection (Rotter et al., 2018). However, considering the above-reported differences in molecular and metabolic responses between plants infected by BNp and FDp, we cannot exclude that FDp-infected plants are more sensitive to virus attack, thus opening the venue for future research studies specifically addressed to explore this response.

The novel vvi_miC64-3p, significantly induced in R (**Figure 4**), is the miRNA^∗^ of vvi_miC64-5p controlling a Polyphenol oxidase II-encoding transcript (*VvPPO*, VIT_10s0116g00560) tied to lignin biosynthesis and to resistance responses to fungi and bacteria (Harm et al., 2011). The vvi_miC64-5p was overall more abundant and followed a different trend of accumulation than vvi_miC64-3p. In particular, the over-accumulation of vvi_miC64-5p in R and FD samples occurred in parallel with the downregulation of *VvPPO* transcription (**Figure 4**). This downregulation may negatively influence lignification of infected tissues, thus representing another example of down-regulation of a pathway involved in response to pathogens.

Although mechanisms of plant response to the pathogen still require further deepening, even less is known on the molecular bases driving the phenomenon of symptom recovery in grapevine infected by FD. Recovered grapevines are asymptomatic and phenotypically similar to H plants, and the expression levels of miRNAs generally showed a close relationship between R and H categories (**Figure 2**). However, for some combinations of miRNAs/targets, this relationship was not observed. For example, *VvCHL* and *VvPSBx*, targeted by vvi-miR2950-5p and vvi-miR169, respectively, were downregulated in R suggesting a reduced photosynthetic efficiency or a not complete recovery of the gas exchange performances in these plants, as previously reported (Vitali et al., 2013). In addition, the lower levels of JA in R grapevines, associated to the modulation of the vvi-miR319/*VvTCP* complex, point out that the recovery condition might be related to repression of JA signaling pathways even after 2 years from the disappearance of FDp. Taken together, these results suggested the persistence for long time of a “molecular memory” of the former phytoplasma infection; further investigations are needed to verify whether this “memory” could be effective to prime the recovered plants from new FDp infection events.

## Conclusion

In this work, we univocally cataloged for the first time all novel miRNAs identified so far in grapevine by producing a single database, miRVIT, with the final aim to put order in an intriguing research field that has been developing and evolving continuously over recent years. In addition to conserved miRNAs, some novel miRNAs detected by several authors (vvi_miC64, vvi_miC137) were shown to be *bona fide* miRNAs, spread among different genotypes, potentially regulated by diverse environmental conditions and likely playing important biological functions that should be further investigated in the future through *ad hoc* functional studies. miRVIT will integrate several existing bioinformatics resources designed for transcriptome, small RNAs and functional analysis in grapevine (Grimplet et al., 2009; Dereeper et al., 2011; Wong et al., 2013; Belli Kullan et al., 2015; Pulvirenti et al., 2015; Moretto et al., 2016). We also demonstrated that the application of miRVIT to the analysis of miRNAs from FDp-infected grapevines was effective for pinpointing complex interactions among miRNAs and related targets specifically linked to disease evolution. In particular, we evidenced that inhibition of cell development and photosynthetic processes (vvi-miR156/*VvSPL*, *VvAGO10*/vvi-miR166/*VvPHB*, and vvi_miC137-3p/*VvPSI*) could be finely tuned by miRNAs in infected plants together with regulation of the cross-talk between JA signaling pathways (vvi-miR319/*VvTCP* and vvi-miR167/*VvARF*) and disease resistance response (vvi-miR482/*VvMLA10*, vvi_miC1031-5p/*VvAGO2*, and vvi_miC64-5p/*VvPPO*).

## Author Contributions

GG, IP, and CM conceived the study. GG produced the miRVIT database. GB developed the database platform. SA performed the bioinformatic analyses. WC and CP performed most of the molecular analyses and elaborated the corresponding data. PB, MR, SP, and IP helped with the molecular analyses and complemented the writing. GG wrote the manuscript with the help of WC, CP, and IP. All authors read and approved the manuscript.

## Conflict of Interest Statement

The authors declare that the research was conducted in the absence of any commercial or financial relationships that could be construed as a potential conflict of interest.

## Footnotes

1 http://genomes.cribi.unipd.it/grape/

2 http://genomes.cribi.unipd.it/DATA/V1/

3 https://www.blast2go.com/

4 http://unafold.rna.albany.edu

5 https://sourceforge.net/projects/bowtie-bio/files/bowtie/1.1.2/

6 http://compbio.dfci.harvard.edu/compbio/tools/mev
