# Supplementary material for: miRVIT: A Novel miRNA Database and Its Application to Uncover Vitis Responses to Flavescence dorée Infection
Source: Front Plant Sci. 2018 Jul 17;9:1034. doi: 10.3389/fpls.2018.01034 (PMC6057443; doi:10.3389/fpls.2018.01034)
Supplement: Supplementary file 11 [file Image_1.PDF]

Supplementary Figures

```
c124028_Bester2017b        --------------------------------TGTTTTGTTGCTGGTCATCTAGTCATTGTTAGTCATGTTTAACGCTCATGGCAGGATGATTAGATGATCATCAACAAACACAT----------------------------------
VV-MIR072_Wang2011         --------------GTTTTGCAGCTATGAATATGTTTTGTTGCTGGTCATCTAGTCATTGTTAGTCATGTTTAACGCTCATGGCAGGATGATTAGATGATCATCAACAAACACATTCAGGATAATGCATCAC-----------------
va-miR023_Wang2012         -------------TGTTTTGCAGCTATGAATATGTTTTGTTGCTGGTCATCTAGTCATTGTTAGTCATGTTTAACGCTCATGGCAGGATGATTAGATGATCATCAACAAACACATTCAGGATAATGCATCAC-----------------
Vvi-miRC71_Han2014         --------------GTTTTGCAGCTATGAATATGTTTTGTTGCTGGTCATCTAGTCATTGTTAGTCATGTTTAACGCTCATGGCAGGATGATTAGATGATCATCAACAAACACATTCAGGATAATGCATCAC-----------------
novel_mir_21_Sun2015       --------------GTTTTGCAGCTATGAATATGTTTTGTTGCTGGTCATCTAGTCATTGTTAGTCATGTTTAACGCTCATGGCAGGATGATTAGATGATCATCAACAAACACATTCAGGATAATGCATCAC-----------------
vvi-miRC827_BelliKullan2015---------TATGTGTTTTGCAGCTATGAATATGTTTTGTTGCTGGTCATCTAGTCATTGTTAGTCATGTTTAACGCTCATGGCAGGATGATTAGATGATCATCAACAAACACATTCAGGATAATGCATCACACCAC------------
vvi-miRC827_Pinto2016      ---------TATGTGTTTTGCAGCTATGAATATGTTTTGTTGCTGGTCATCTAGTCATTGTTAGTCATGTTTAACGCTCATGGCAGGATGATTAGATGATCATCAACAAACACATTCAGGATAATGCATCACACCAC------------
n221_Pagliarani2017        AGGGTCTAGTATGTGTTTTGCAGCTATGAATATGTTTTGTTGCTGGTCATCTAGTCATTGTTAGTCATGTTTAACGCTCATGGCAGGATGATTAGATGATCATCAACAAACACATTCAGGATAATGCATCACACCACGGCTGTGTCTCT
c193477_Bester2017         ---------------------------------GTTTTGTTGCTGGTCATCTAGTCATTGTTAGTCATGTTTAACGCTCATGGCAGGATGATTAGATGATCATCAACAAACACATT---------------------------------
c101040_Bester2017b        ----------------------------------TTTTGTTGCTGGTCATCTAGTCATTGTTAGTCATGTTTAACGCTCATGGCAGGATGATTAGATGATCATCAACAAACACAT----------------------------------
c129481_Bester2017b        ----------------------------------TTTTGTTGCTGGTCATCTAGTCATTGTTAGTCATGTTTAACGCTCATGGCAGGATGATTAGATGATCATCAACAAACACATT---------------------------------
c125997_Bester2017b        ----------------------------------TTTTGTTGCTGGTCATCTAGTCATTGTTAGTCATGTTTAACGCTCATGGCAGGATGATTAGATGATCATCAACAAACACATT---------------------------------
Vvi-miRC71_Zhao2017        --------------GTTTTGCAGCTATGAATATGTTTTGTTGCTGGTCATCTAGTCATTGTTAGTCATGTTTAACGCTCATGGCAGGATGATTAGATGATCATCAACAAACACATTCAGGATAATGCATCAC-----------------
vvi-miRn001_Snyman2017     AGGGTCTAGTATGTGTTTTGCAGCTATGAATATGTTTTGTTGCTGGTCATCTAGTCATTGTTAGTCATGTTTAACGCTCATGGCAGGATGATTAGATGATCATCAACAAACACATTCAGGATAATGCATCACACCACGGCTGTGTCTCT
vvi_miC132                 ----------------------------------TTTTGTTGCTGGTCATCTAGTC-----------------------------------TTAGATGATCATCAACAAACA-------------------------------------
                                                             **********************                                   *********************
```

Figure S1. Alignment by Clustal W (http://www.genome.jp/tools-bin/clustalw) of the precursors of novel miRNA vvi_miC132 identified in 11 different works.

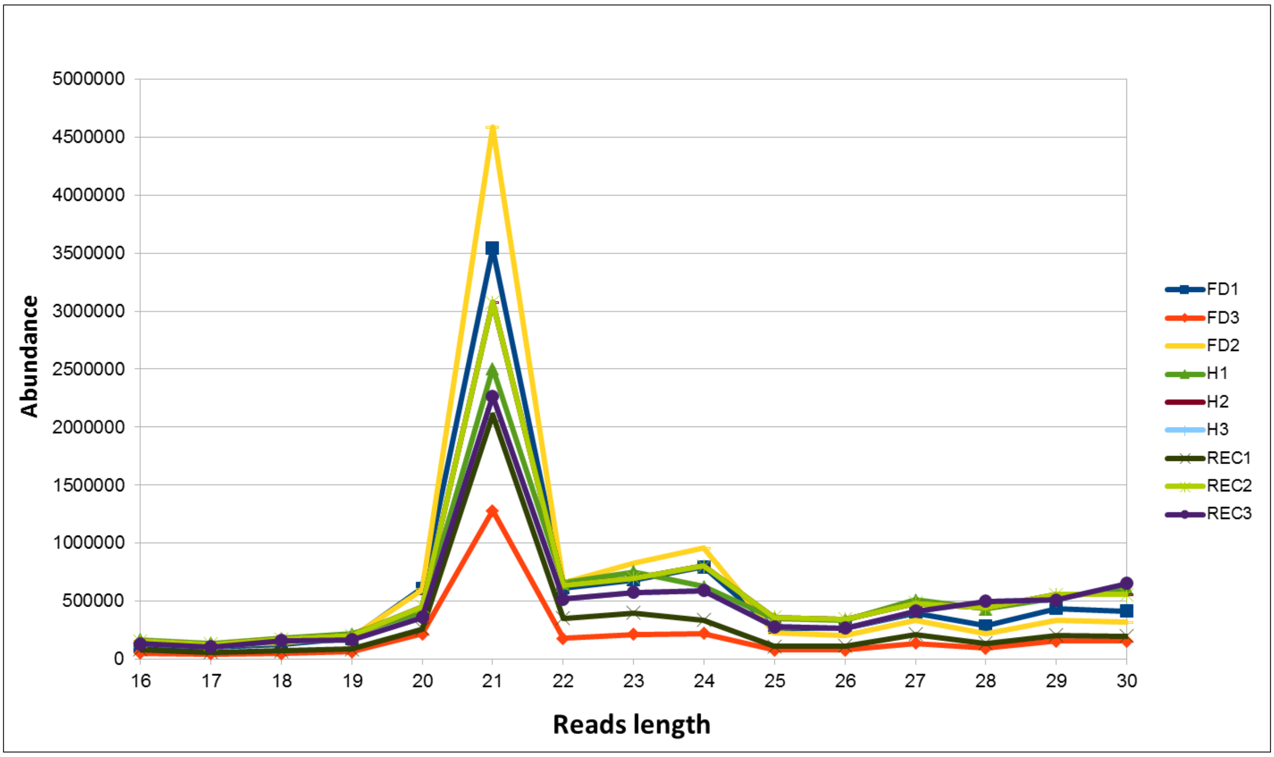

Figure S2. Read length distribution of short RNA sequences from nine libraries of *Vitis vinifera* cv. Barbera leaf midribs collected from FDp-infected (FD), recovered (R) and healthy (H) plants.

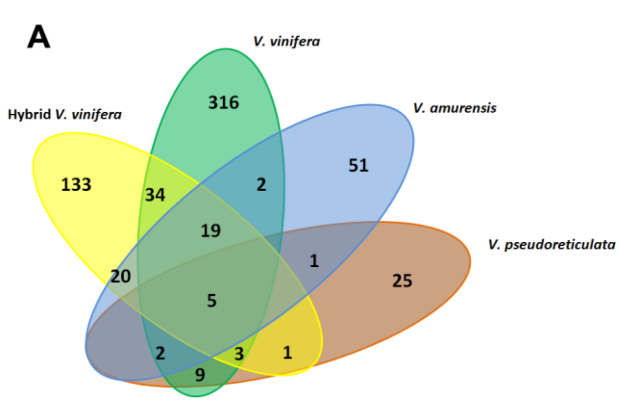

Figure S3. A) Venn diagram displaying the distribution of the 621 novel miRNAs 20–22 nt long based on the genotype in which the miRNAs were originally identified; B) the genotype-based distribution of the novel miRNAs detected in leaf midribs of 'Barbera'.

Figure S4. Predicted secondary structures of putative novel miRNAs identified for the first time in 'Barbera' leaf midribs ('Barbera'-novel miRNAs). Hairpin secondary structures were obtained using MFOLD version 2.3 (http://unafold.rna.albany.edu/?q=mfold). The miRNA and miRNA* sequences are indicated in red and blue, respectively.

vvi_miC134_V1-3p

Hairpin:
CAATTGAGGCTTTAGTTTTTAAATTGAAGCTGCAGTTGCTAAGTGAGGTTTCATTTTTTA
ATTGAAGCCTCAATTGACAACTGAGGCTTCATTTTTGAACTAAAGCCTCAATTG

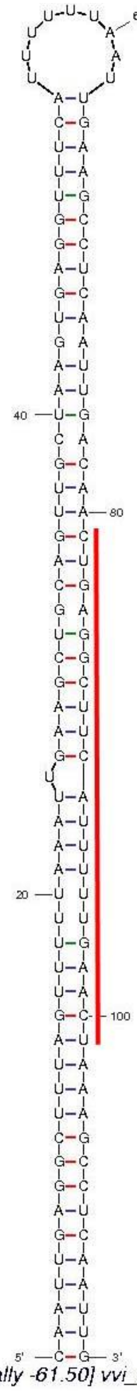

dG = -61.50 [Initially -61.50] vvi_miC134_V1-3p

vvi_miC134_V1b-3p

Hairpin:
CAACTGAGACTTCATTTTTTAAGTGAAGCCTCAATTGACAATTGTGACTTTATTTTTTAA
CTGAAACCTCAATTGATAACTGAGGCTTCATTTTTGAACTGAAGCTGTAGTTG

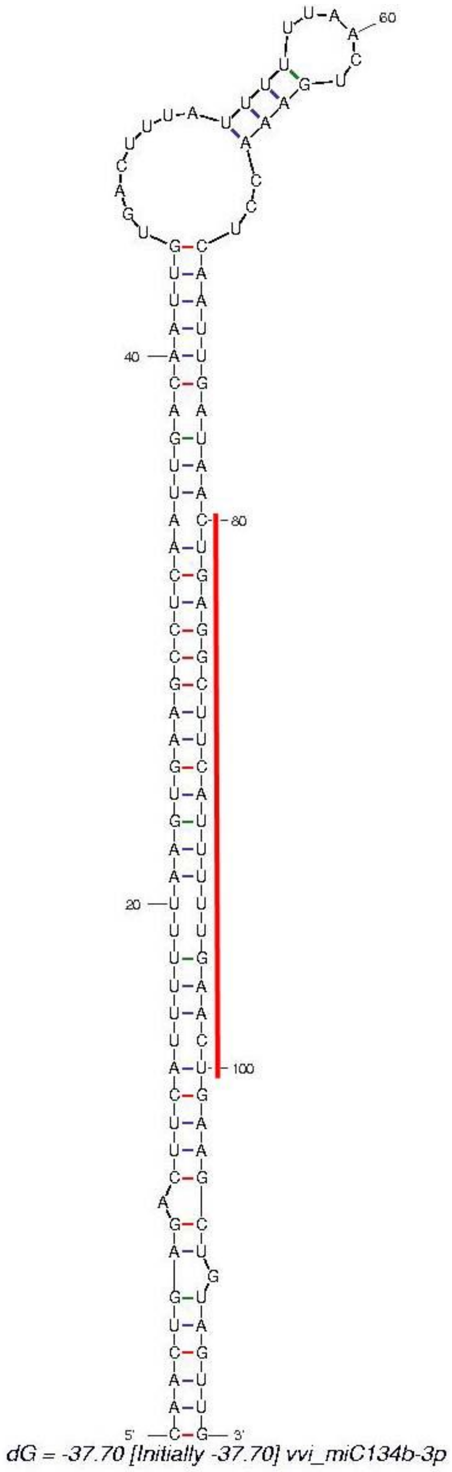

vvi_miC443_V1-3p / vvi_miC443_V1-5p

Hairpin:
GGATTCTCAGAGGCAATATTACAGAGCCATTTTGGCATTCTGTCCACCTCCATATATAC
CAATTCTTCTGAATTGGCGCCCACAAGCGTTTGGAGGCGGCCAGGATGCCAAATTGGC
TCTGTAAATTTCTCTCTGAGAAGCC

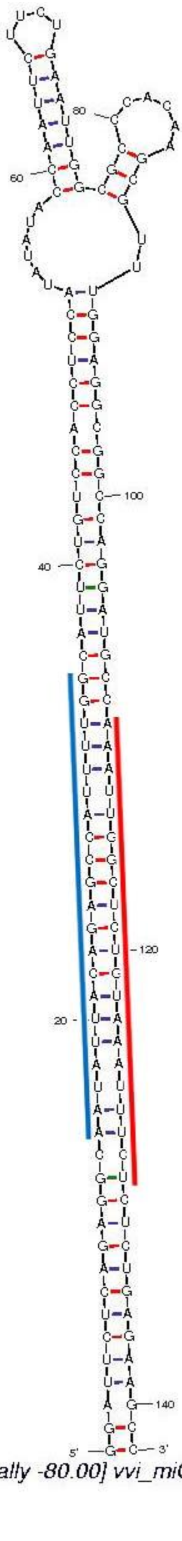
dG = -79.68 [Initially -80.00] vvi_miC443_V1-3p

vvi_miC593_V1-5p / vvi_miC593_V1-3p

Hairpin:
ATGTATATATTTTGAAGAAGGTTTTGTTGCCTTTTAATGGTGAACCAAATAACTCTGGT
TATGTTGGTCCATATGTCACAATCATATAGAGAGCATCATAACCAGAGTTCTTTAGTTG
ACGATTCAGAAGTAATGAAAACTTTTTCAAAATATATATAT

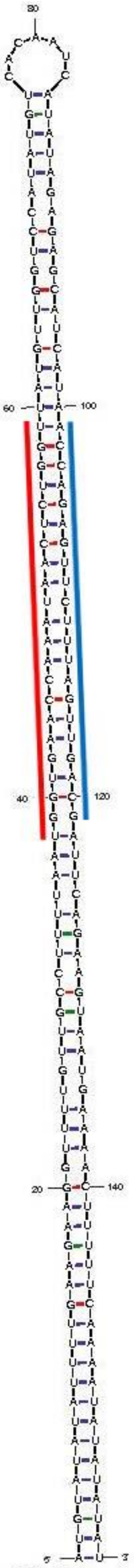

dG = -63.60 [Initially -63.10] vvi_miC593_V1-3p

vvi_miC597-3p

Hairpin:
AGGACCTTGGTCTTGAGTTTGAATCCTGGGGAGGCCACTTTGGGTCGCTTTGGGACTAA
AGTGACATCCCTAGGATTCGAATTGACGTCCCCAGGATTCGAACTCAAGACCAAAGCC
T

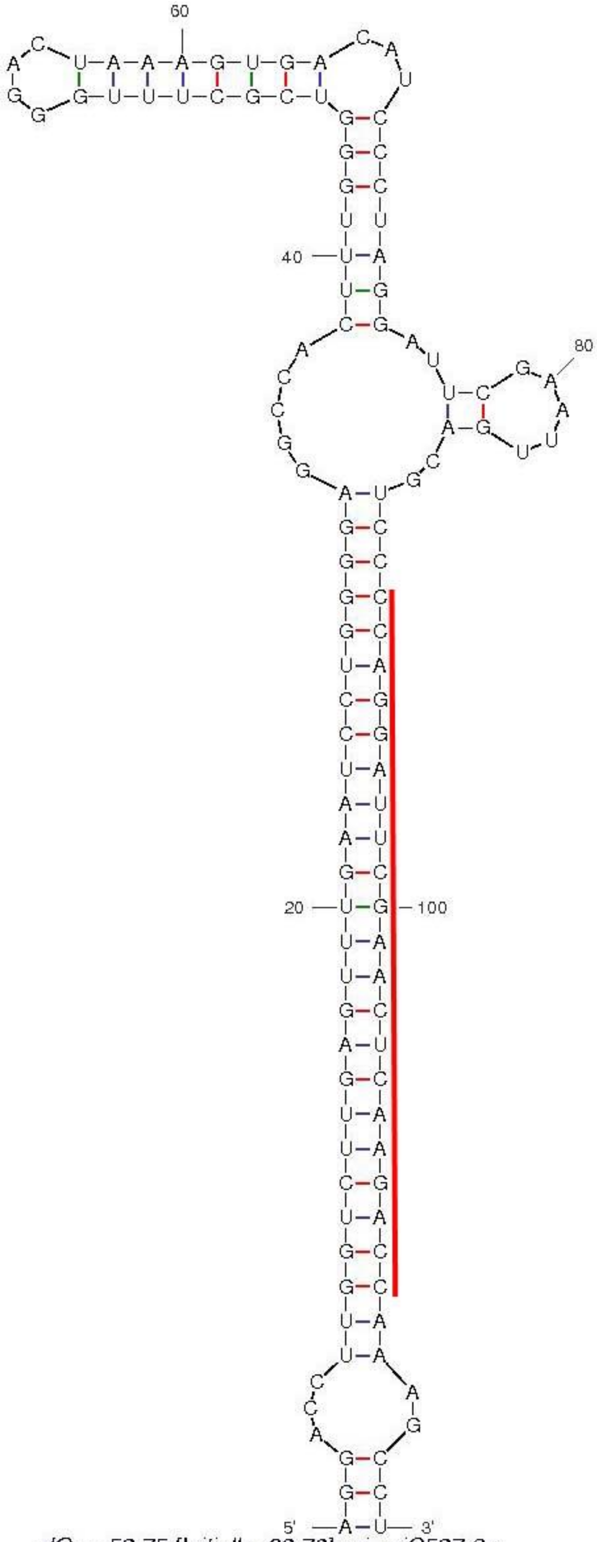

dG = -59.75 [Initially -62.70] vvi_miC597-3p

vvi_miC605-3p

Hairpin:
GTTTGTACACATCTTCTCTTTGTTTCCTTAGCCTCTTGAGTTACAGACAGAGTTTGAGAG AGGCTCGGTGAAATAGACATGTCTGTGAAGATGCAGAC

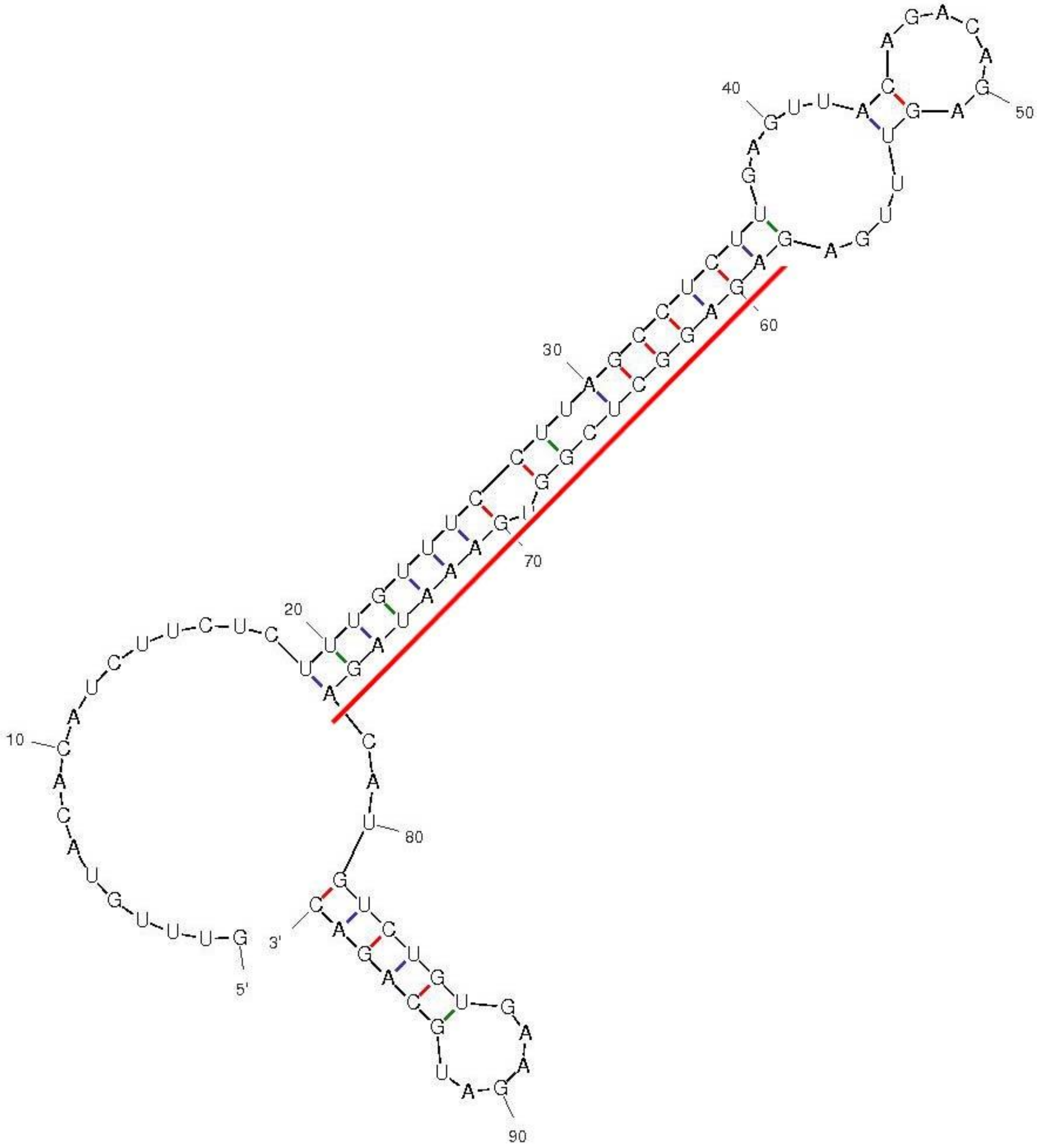

dG = -27.10 [Initially -27.10] vvi_miC605-3p

vvi_miC606-3p

Hairpin:
AGCTCGTAATGAGGGACGTCATTTTGCTTATGAGGGTAATGAAATTATCCATGCAGCTAGCAAATGGAGTCCTGAACTAGTTGCT

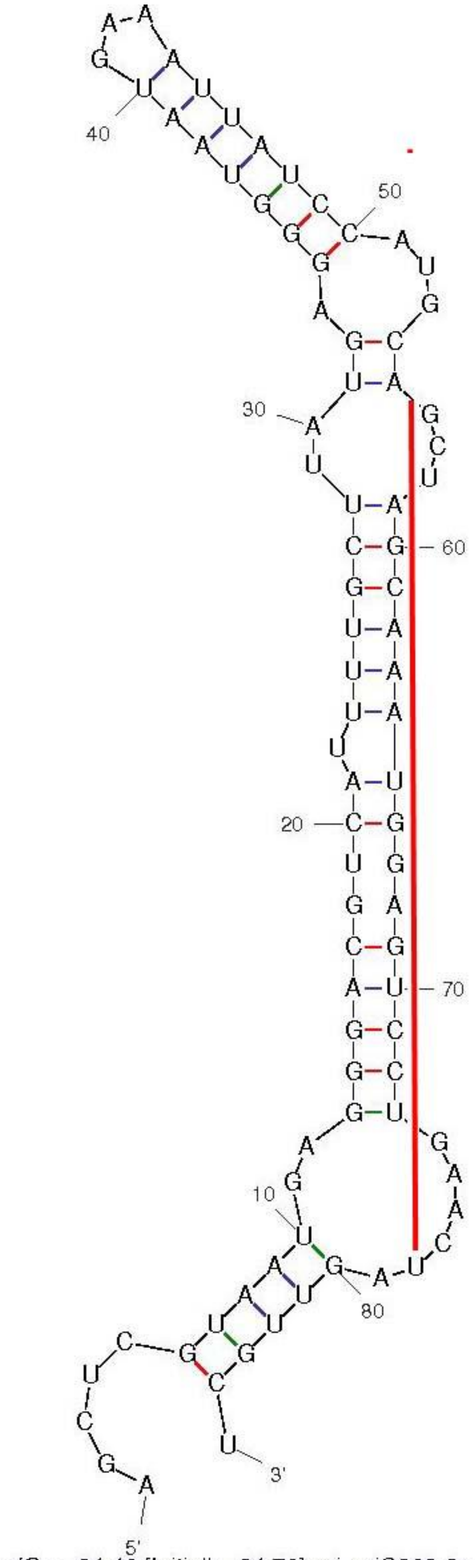

dG = -24.40 [Initially -24.70] vvi_miC606-3p

vvi_miC614-5p

Hairpin:
AGAGTGTCTTTGGTAGTGATTTTAGGAAGTGTTTCTGGGCTTTATAACACTTGGAAATT
TTCATCCTTCAAATATTATAAAGCCTAGAAATACTTCCTATAATCACTGCCAAATGCAC
TCT

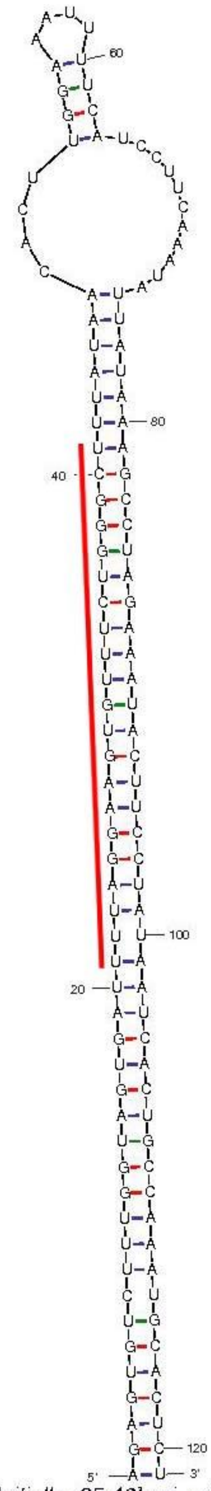

dG = -65.90 [Initially -65.40] vvi_miC614-5p

vvi_miC617-3p

Hairpin:
AGAACATAGGAGTAGGGTGTGTTGAGACGTCATTTGTTTTGTCTGTACTCATAGCGACC
AGATTTATTTTTGGCACCAGAGCAAAAAATAAATTTGTTCGCTATGAGCACGAACAAA
ACAAATGACGTCCCAACAAAAACGTCGGCCTTTTGTGCTCT

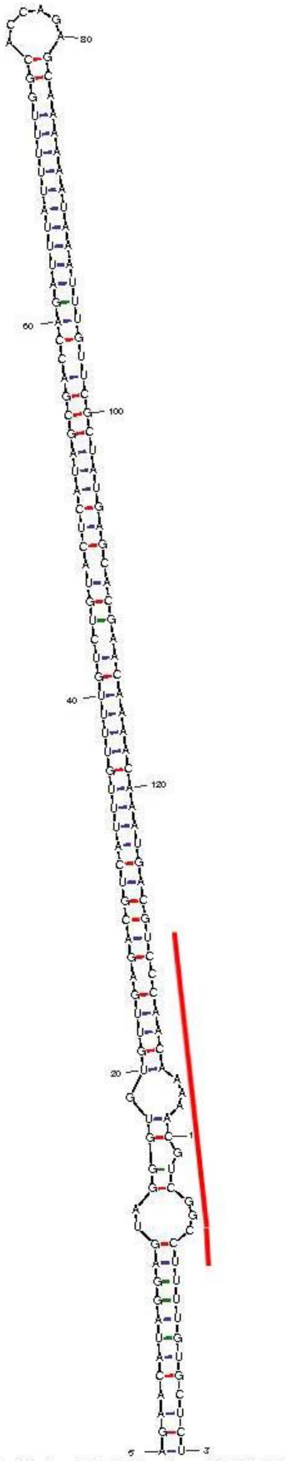

dG = -74.30 [Initially -73.80] vvi_miC617-3p

vvi_miC620-3p

Hairpin:
GGCTCAGGGAAGAGGCAGGGCGGAAGCTGGTGGTTGAGTGTACCGGTGAAGGTATCG
TCTTCATTGAAGCCGATGCCGACGTTACGCTTGAGCAGTTCGGCGATGCTCTTCAGCCT
CCATTCCCGGGCT

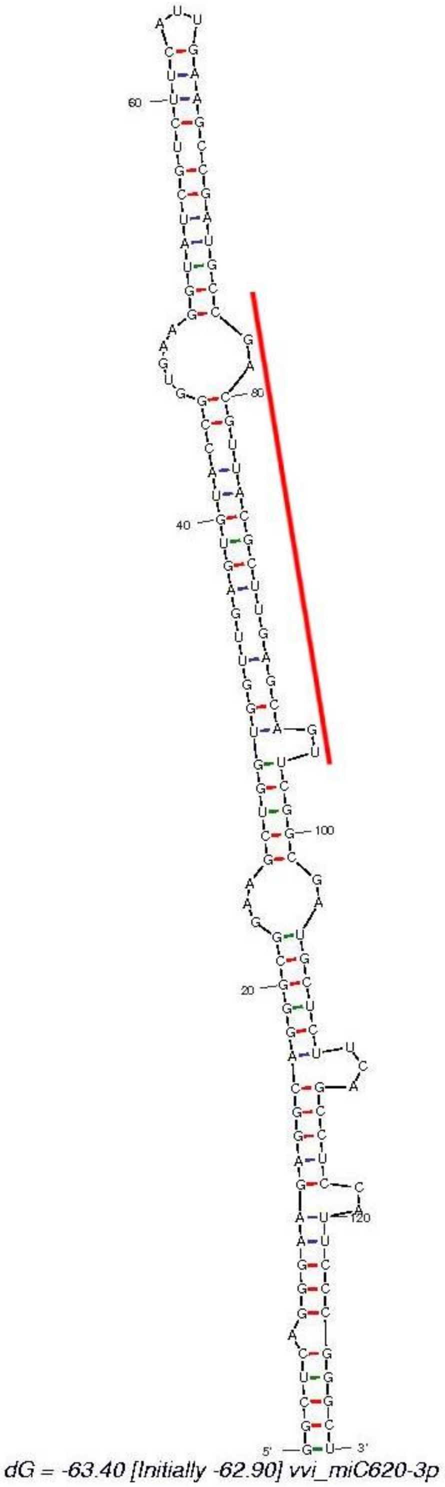

dG = -63.40 [Initially -62.90] vvi_miC620-3p

vvi_miC630-3p

Hairpin:
ATTTTTTCTTGCATTTTCCTTCAATTTTTTTGGGAACCAAATATAGCCAAAAAGAAGAA
AGAAAAAAAAAAAAAAAAAGAAAAAAAAAAGAAAAAGAAAAAATAGATTTAGGTTATA
CTTGGTTCCTGGAAAATTTGAGGGAGAATATGAGAAAAAAT

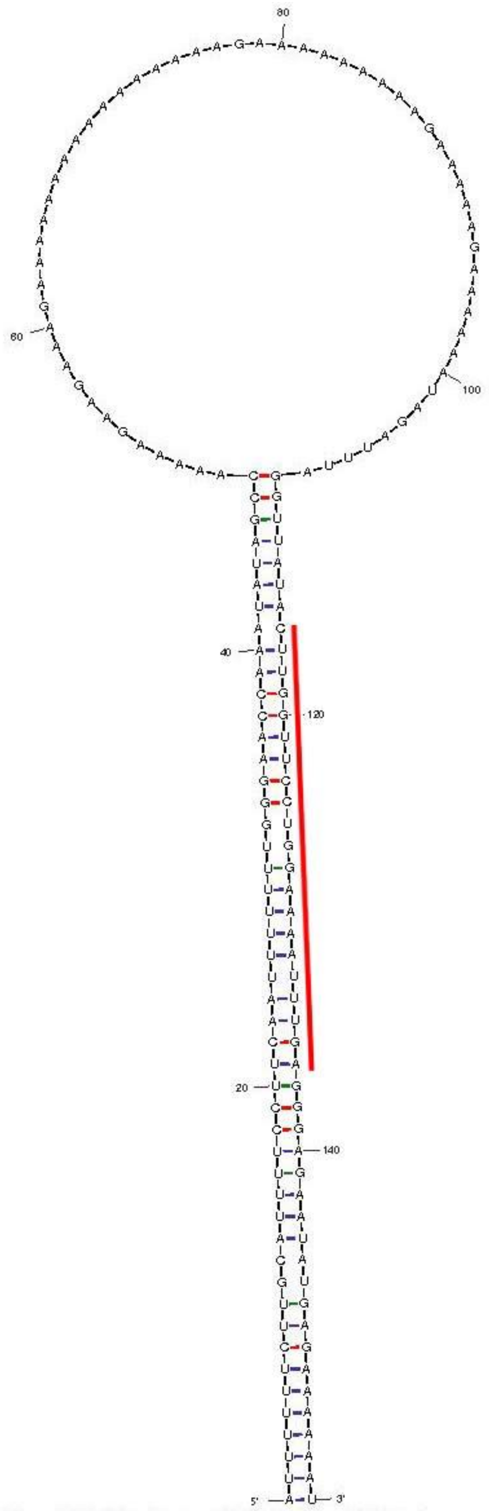

dG = -50.76 [Initially -50.25] vvi_miC630-3p

vvi_miC631-3p

Hairpin:
CCATGATCATAGACCAACCTCAATGATCGTAGACCAACCTCATTCTCATTTTGGTTTTA
ATATTATGTTATGGATCTTGGTTTGTGCTTTATGG

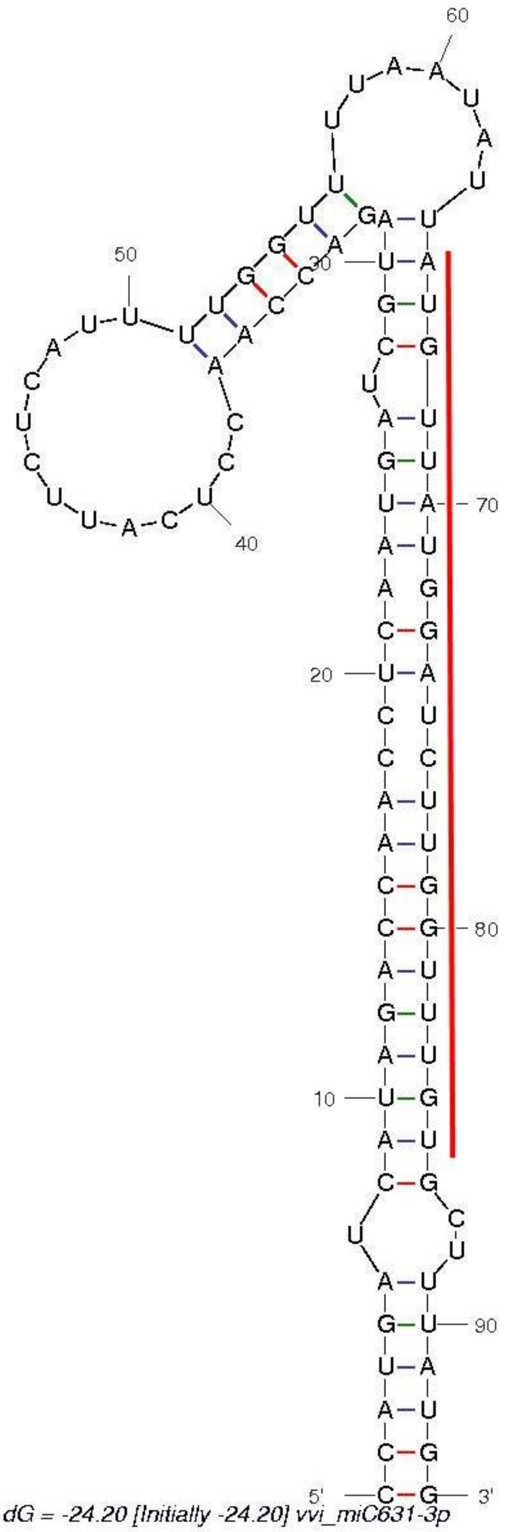

dG = -24.20 [Initially -24.20] vvi_miC631-3p

vvi_miC638-3p

Hairpin:
TAAGGGGGGAGTGTCAAGCCATTAGTGAGATATCACTTTGGAAGAGCTAGAATTCTAACCTTA

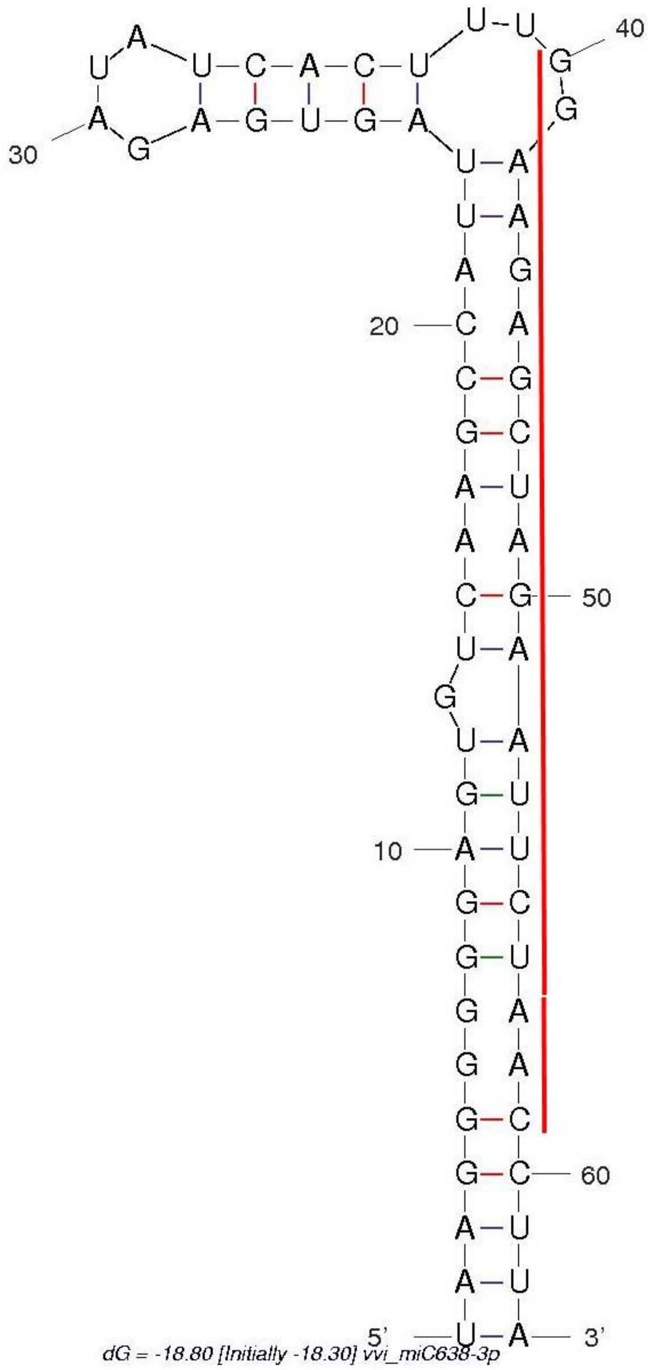

vvi_miC644-3p

Hairpin:
TAGAGATGGAGGACGTGTTGGGCTTAGAGTAAGGGAAGCTGCTCGTGGATGTGGAGG
AGTGGTAGTAGGCCTGGTGGTCGTAGGAGTGGTTGAGCGATCAAAACGGGCCTTCGTC
CTG

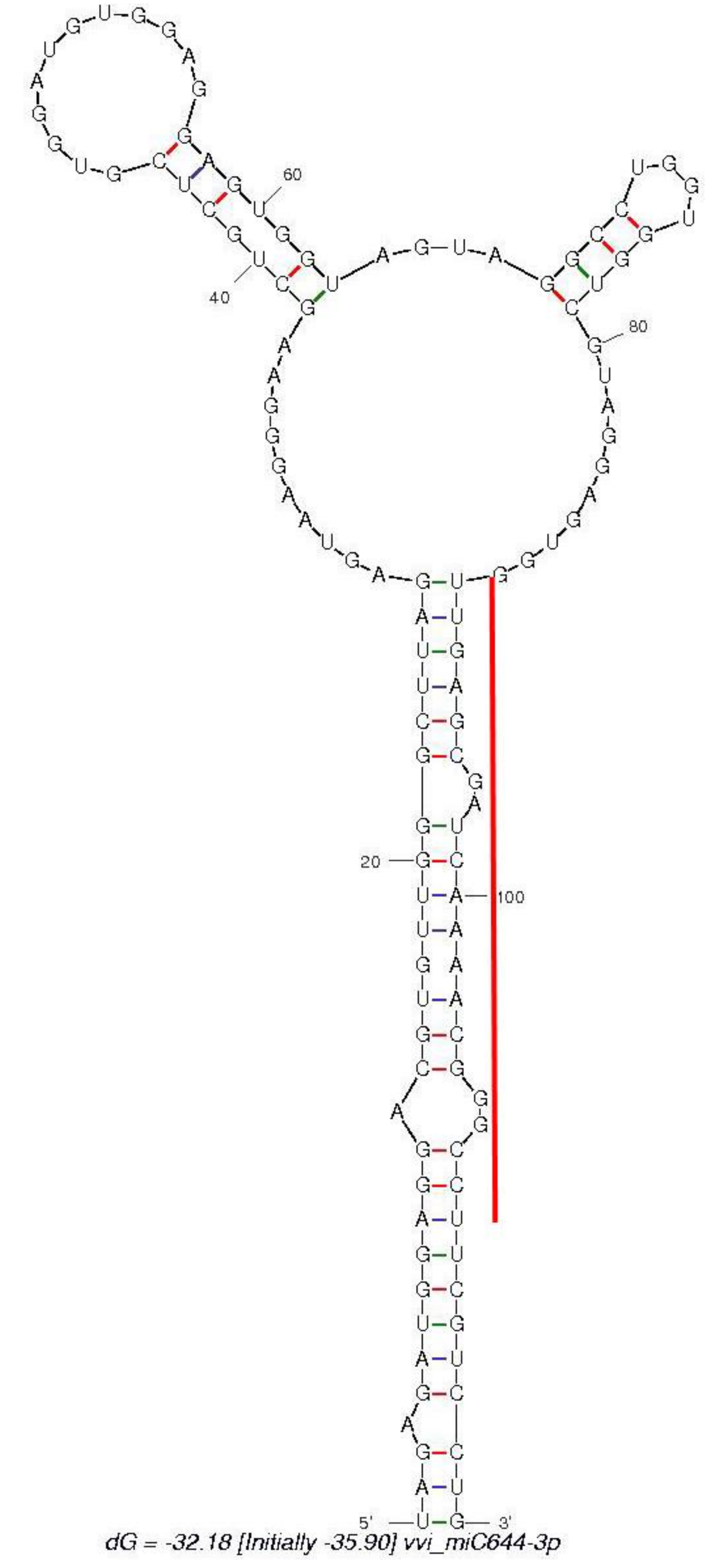

dG = -32.18 [Initially -35.90] vvi_miC644-3p

vvi_miC645-5p

Hairpin:
AGGCACCCCAAGAGGAACCGAAACTAGGACTAAACCTTATGTCCAACCAACTTCTAGT
ACCATCTGGGCTAGTTTGGACATAAGGTTTAGTCTTAGTTTCGATTCCTCCGGGGGACA
CCT

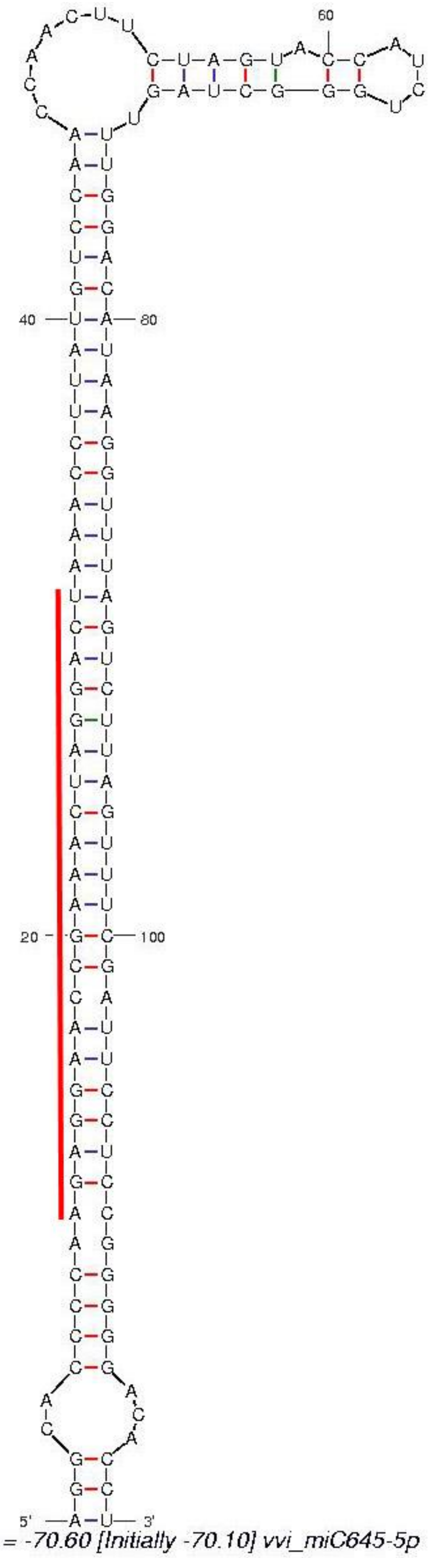

dG = -70.60 [Initially -70.10] vvi_miC645-5p

vvi_miC648-5p

Hairpin:
ATATGAGTTTTAAGCGAGACCATTGTGACTACTCCAGATTTAACTGGGGGATTTCTTCA AAGTCTTTATTGATTTTCATAT

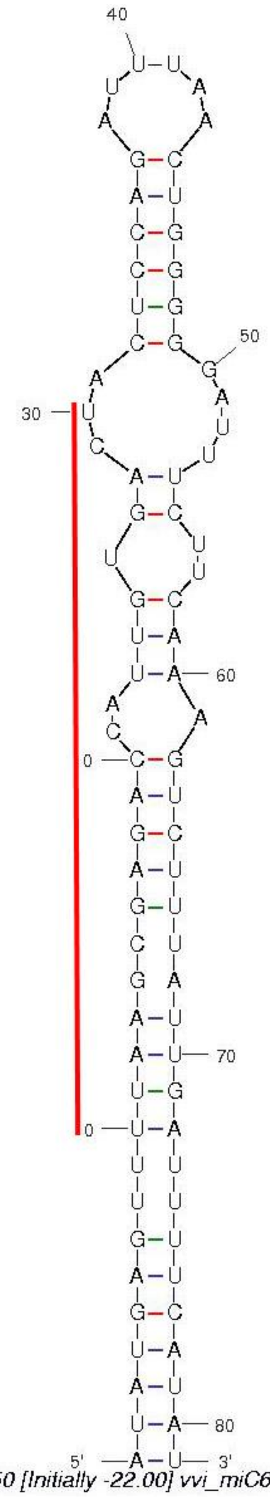

dG = -22.50 [Initially -22.00] vvi_miC648-5p

vvi_miC653-5p

Hairpin:
CCACACAAGATCAACTATGAGCCCCTTGAATGTGGAAGAAGAGGGCCATGAGGGCAAGAGGAGACCCAACTTCCCAGGCCATGAAAGCTTCAAGAGGAGCTCAGTTGATCCTGTTGG

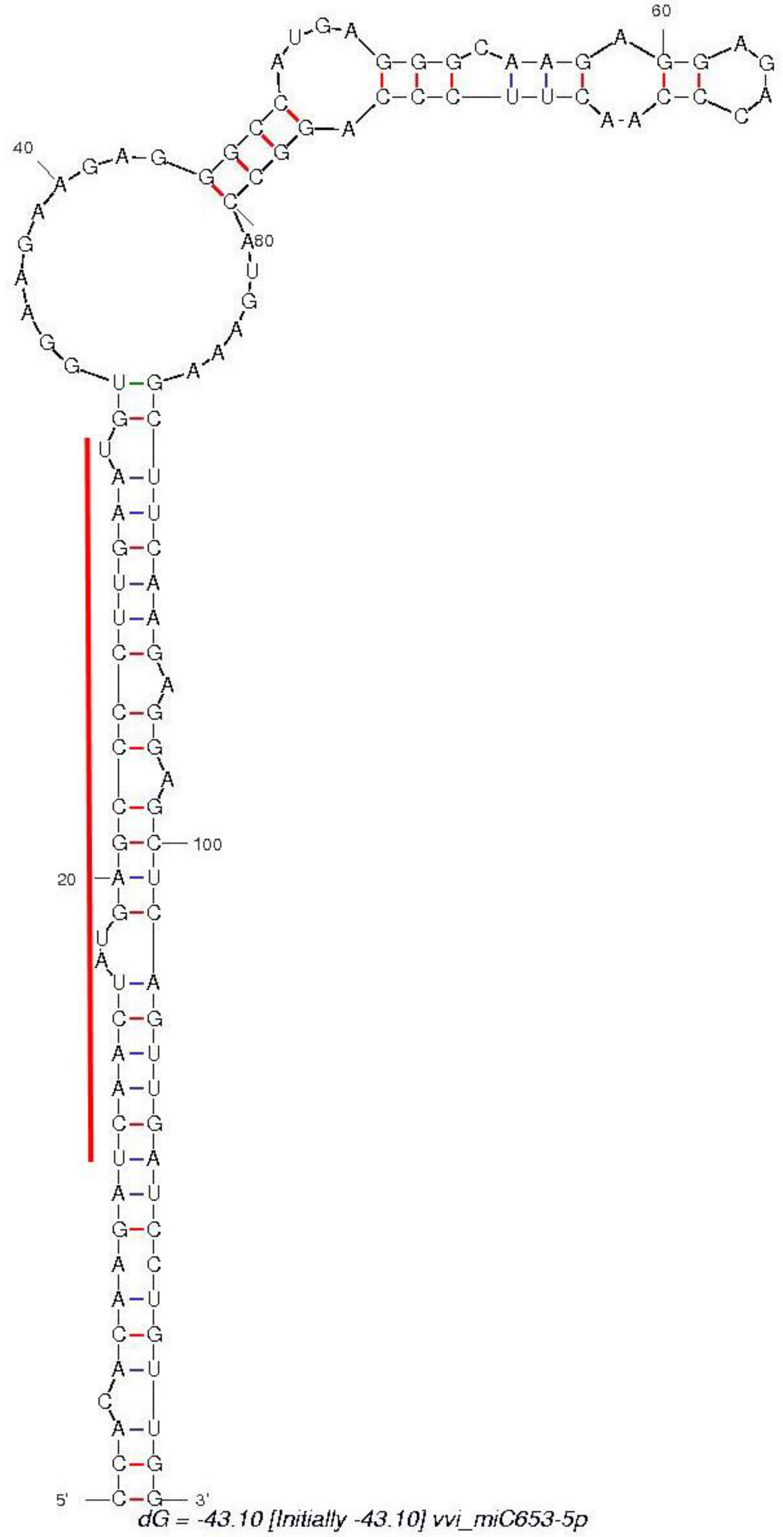

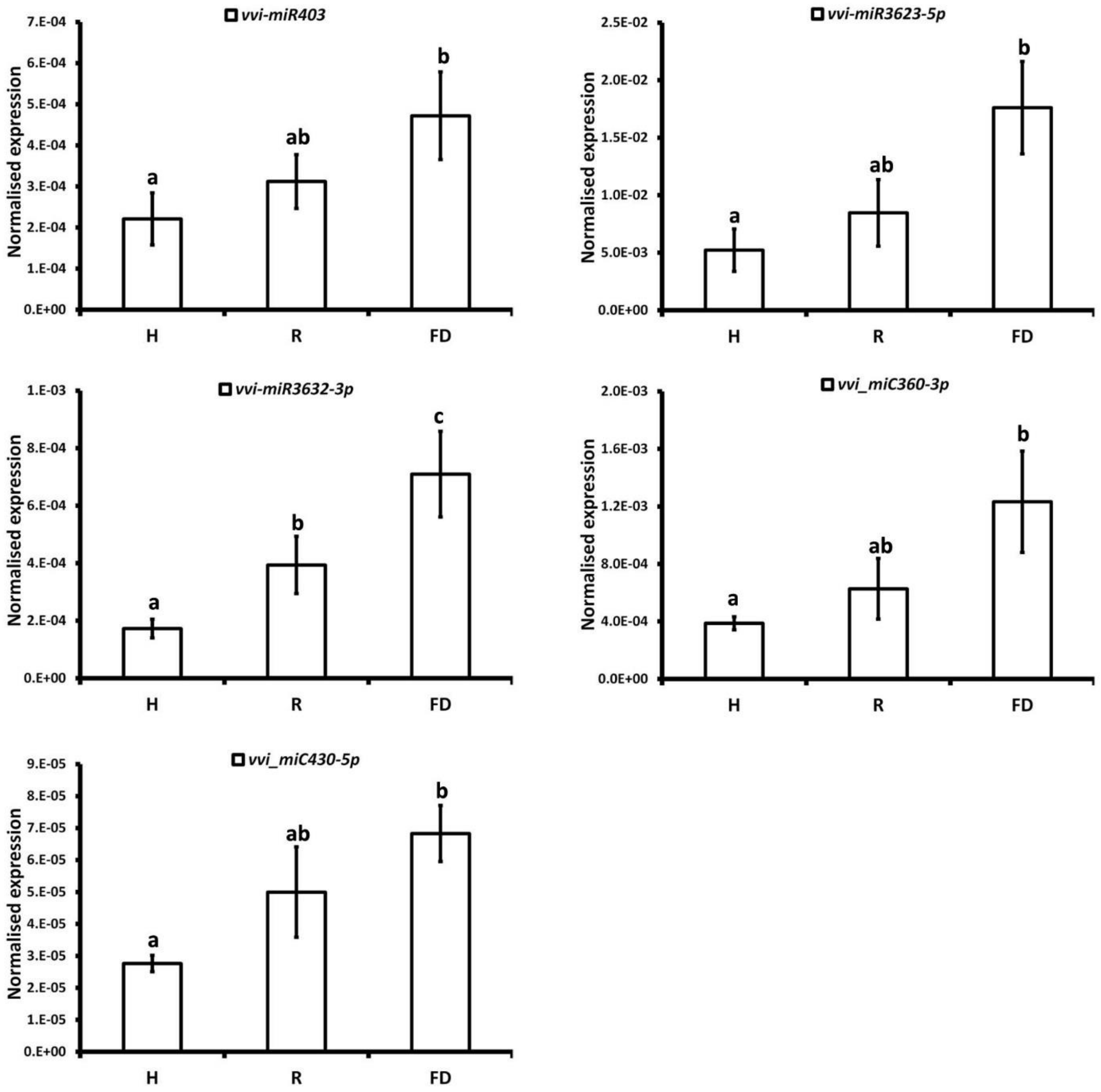

Figure S5. Expression levels of vvi-miR3632-3p, vvi-miR3623-5p, vvi-miR403, vvi_miC360-3p and vvi_miC430-5p in FDp-infected (FD), recovered (R) and healthy (H) leaf midribs of 'Barbera'. qRT-PCR signals were normalised to U6 and 5.8 rRNA. Lowercase letters denote significant differences ($p \leq 0.05$) among miRNAs expression levels tested using Tukey's HSD test. Data are presented as mean ± standard error of five biological replicates (n = 5).

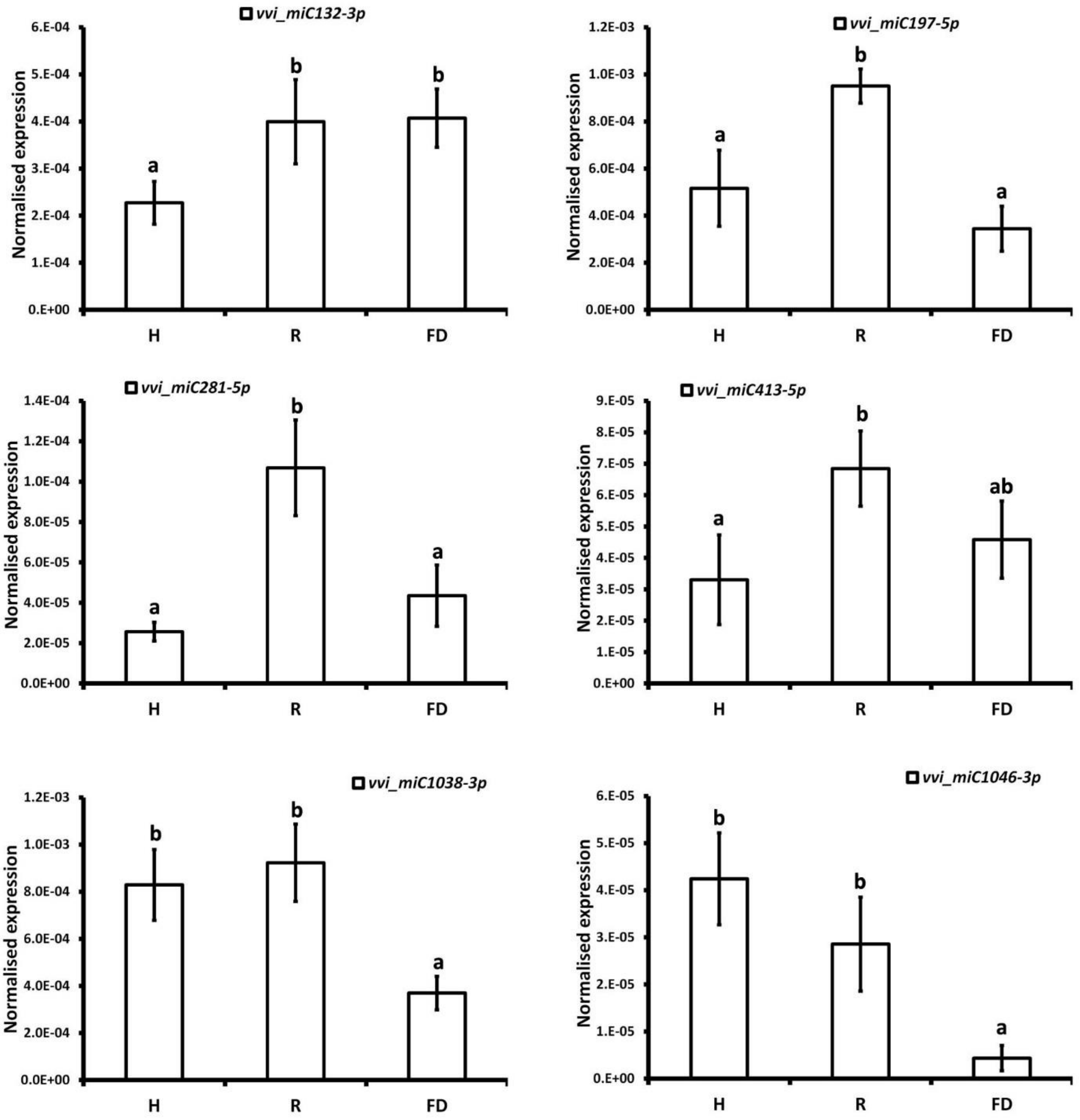

Figure S6. Expression levels of vvi_miC1038-3p, vvi_miC132-3p, vvi_miC197-5p, vvi_miC281-5p, vvi_miC413-5p, and vvi_miC1046-3p in FDp-infected (FD), recovered (R) and healthy (H) leaf midribs of 'Barbera'. qRT-PCR signals were normalised to U6 and 5.8 rRNA. Lowercase letters denote significant differences ($p \leq 0.05$) among miRNAs expression levels tested using Tukey's HSD test. Data are presented as mean ± standard error of five biological replicates (n = 5).

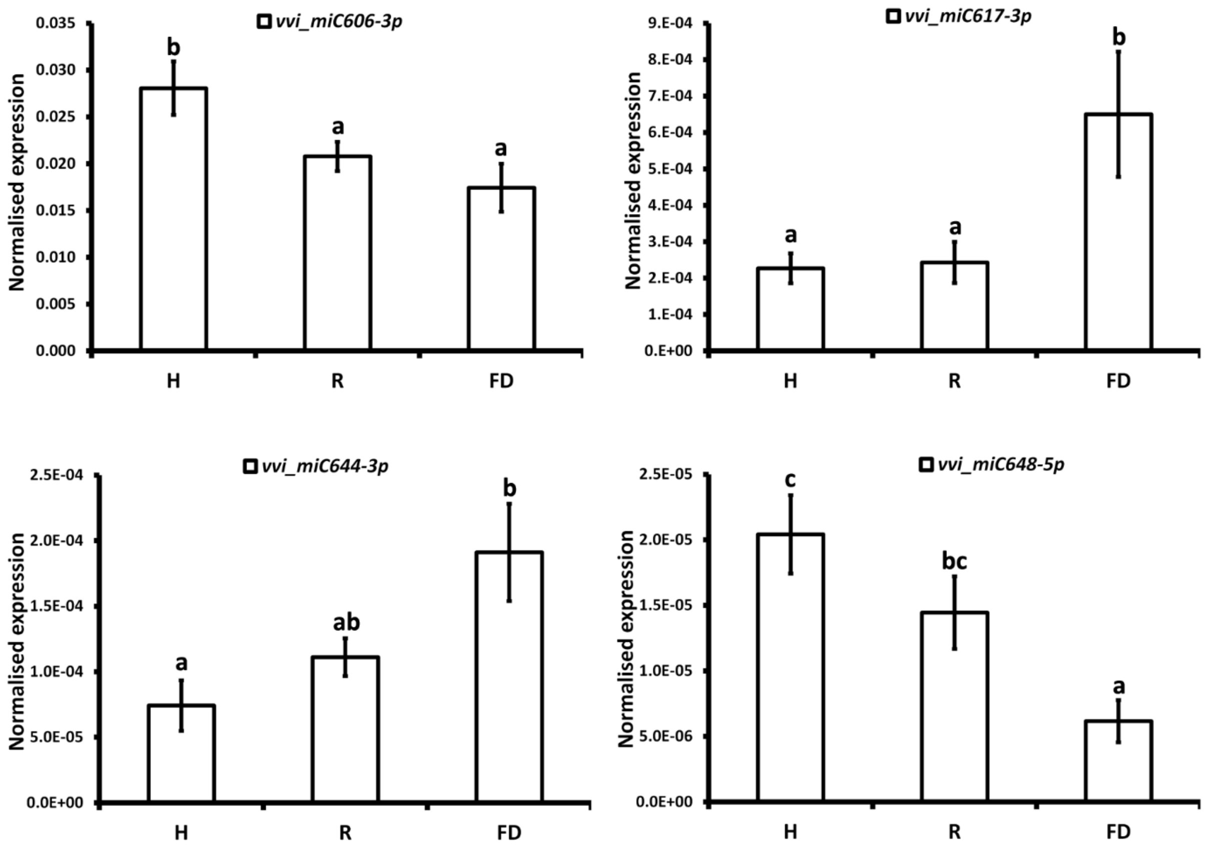

Figure S7. Expression levels of 'Barbera'-novel miRNAs: vvi_miC606-3p, vvi_miC617-3p, vvi_miC644-3p and vvi_miC648-5p in FDp-infected (FD), recovered (R) and healthy (H) leaf midribs. qRT-PCR signals were normalized to U6 and 5.8 rRNA. Lowercase letters denote significant differences ($p \leq 0.05$) among miRNA expression levels tested using Tukey's HSD test. Data are presented as mean ± standard error of five biological replicates ($n = 5$).

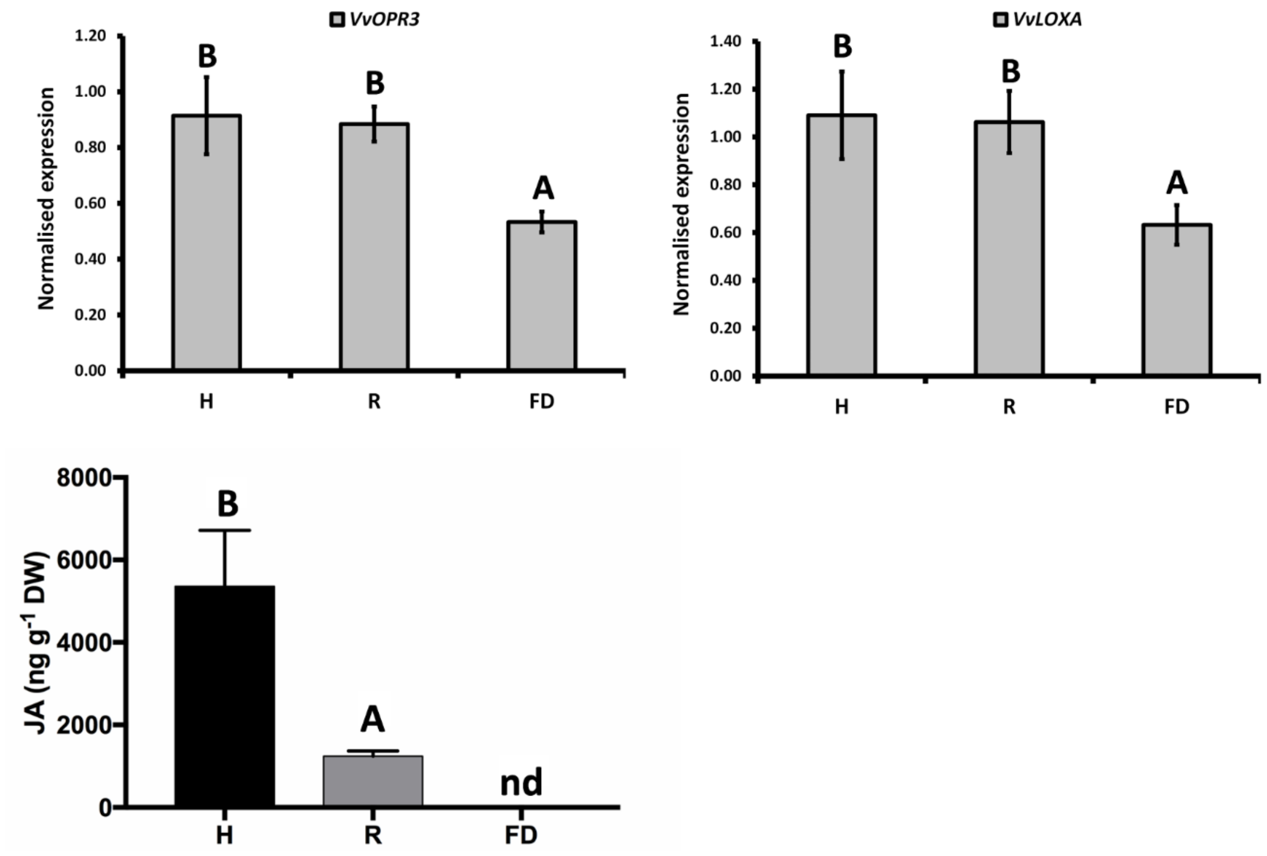

Figure S8. Expression levels of *VvOPR3* (VIT_11s0016g01230), *VvLOXA* (VIT_06s0004g01510) and Jasmonate (JA) quantification in FDp-infected (FD), recovered (R) and healthy (H) leaf midribs of 'Barbera'. qRT-PCR signals were normalised to actin and ubiquitin transcripts. Uppercase letters denote significant differences ($p \leq 0.05$) among miRNAs expression levels or JA levels attested using Tukey's HSD test. Data are presented as mean ± standard error of five biological replicates (n = 5).
